# Physical activity, multimorbidity, and life expectancy: a UK Biobank longitudinal study

**DOI:** 10.1186/s12916-019-1339-0

**Published:** 2019-06-12

**Authors:** Yogini V. Chudasama, Kamlesh K. Khunti, Francesco Zaccardi, Alex V. Rowlands, Thomas Yates, Clare L. Gillies, Melanie J. Davies, Nafeesa N. Dhalwani

**Affiliations:** 1 Diabetes Research Centre, Leicester General Hospital, University of Leicester, Leicester, LE5 4PW UK; 2 National Institute for Health Research (NIHR) Collaboration for Leadership in Applied Health Research and Care - East Midlands (CLAHRC-EM), Leicester Diabetes Centre, Leicester, LE5 4PW UK; 3 NIHR Leicester Biomedical Research Centre, Leicester Diabetes Centre, Leicester, LE5 4PW UK; 4 Alliance for Research in Exercise, Nutrition and Activity (ARENA), Sansom Institute for Health Research, Division of Health Sciences, University of South Australia, Adelaide, SA 5001 Australia; 5 Current address: Real World Evidence, Evidera, London W6 8BJ UK

**Keywords:** Multimorbidity, Co-morbidity, Physical activity, Life expectancy, Mortality, UK Biobank

## Abstract

**Background:**

Multimorbidity is an emerging public health priority. Physical activity (PA) is recommended as one of the main lifestyle behaviours, yet the benefits of PA for people with multimorbidity are unclear. We assessed the benefits of PA on mortality and life expectancy in people with and without multimorbidity.

**Methods:**

Using the UK Biobank dataset, we extracted data on 36 chronic conditions and defined multimorbidity as (a) 2 or more conditions, (b) 2 or more conditions combined with self-reported overall health, and (c) 2 or more top-10 most common comorbidities. Leisure-time PA (LTPA) and total PA (TPA) were measured by questionnaire and categorised as low (< 600 metabolic equivalent (MET)-min/week), moderate (600 to < 3000 MET-min/week), and high (≥ 3000 MET-min/week), while objectively assessed PA was assessed by wrist-worn accelerometer and categorised as low (4 min/day), moderate (10 min/day), and high (22 min/day) walking at brisk pace. Survival models were applied to calculate adjusted hazard ratios (HRs) and predict life expectancy differences.

**Results:**

491,939 individuals (96,622 with 2 or more conditions) had a median follow-up of 7.0 (IQR 6.3–7.6) years. Compared to low LTPA, for participants with multimorbidity, HR for mortality was 0.75 (95% CI 0.70–0.80) and 0.65 (0.56–0.75) in moderate and high LTPA groups, respectively. This finding was consistent when using TPA measures. Using objective PA, HRs were 0.49 (0.29–0.80) and 0.29 (0.13–0.61) in the moderate and high PA groups, respectively. These findings were similar for participants without multimorbidity. In participants with multimorbidity, at the age of 45 years, moderate and high LTPA were associated with an average of 3.12 (95% CI 2.53, 3.71) and 3.55 (2.34, 4.77) additional life years, respectively, compared to low LTPA; in participants without multimorbidity, corresponding figures were 1.95 (1.59, 2.31) and 1.85 (1.19, 2.50). Similar results were found with TPA. For objective PA, moderate and high levels were associated with 3.60 (− 0.60, 7.79) and 5.32 (− 0.47, 11.11) life years gained compared to low PA for those with multimorbidity and 3.88 (1.79, 6.00) and 4.51 (2.15, 6.88) life years gained in those without. Results were consistent when using other definitions of multimorbidity.

**Conclusions:**

There was an inverse dose-response association between PA and mortality. A moderate exercise is associated with a longer life expectancy, also in individuals with multimorbidity.

**Electronic supplementary material:**

The online version of this article (10.1186/s12916-019-1339-0) contains supplementary material, which is available to authorized users.

## Background

The average life expectancy continues to increase in most of developed countries; however, this longevity does not necessarily translate in better health [1]. The ageing phenomenon has led to a substantial increase in chronic conditions, which consequently result in a rising prevalence of multimorbidity, most commonly described as the presence of two or more long-term conditions [2–4]. Multimorbidity has been linked to poor prognosis, lower quality of life [5], increased health care costs, and the risk of premature death [6]. Management of multimorbidity is a complex process, which recently has become an emerging priority for public health care professionals and health care systems [7–9]. Physical activity (PA) has been recommended as one of the main lifestyle behaviours in the management of several chronic conditions worldwide [10–12]. Yet, it is not clear whether and to what extent the benefits of PA apply to people with multimorbidity [9].

To our knowledge, only two studies to date have assessed the relationship between PA and mortality in people with multimorbidity, with inconsistent findings. The first study showed that, in people with multimorbidity, high PA levels were related to a significantly lower risk of mortality, compared to being physically inactive [13]. Conversely, the second study demonstrated that the risk of mortality was only significantly reduced when engaging in extremely high levels of PA (≥ 8000 moderate-vigorous PA MET-min/month), which may not be feasible for individuals with multimorbidity [14]. Moreover, both studies used self-reported PA measures which have several limitations [15]. For this reason, there is a need for accurate and reliable measures of PA [16, 17]. Objective PA is capable of capturing precise estimates of energy expenditure at different activity intensities, in particular recording light PA [18]. Yet, to date, no study has examined the association between objective PA and mortality in people with multimorbidity. Furthermore, the life expectancy according to PA levels and multimorbidity status remains not well determined.

We aimed to investigate the association between PA and all-cause mortality and to estimate the effects of PA on life expectancy in people with and without multimorbidity, using both subjective and objective measures of PA in a large contemporary cohort from the UK.

## Methods

### Data source and study population

This research has been conducted using the UK Biobank Resource (Application Number 14146). UK Biobank is a prospective study designed to improve the prevention, diagnosis, and treatment of chronic diseases in people aged between 38 and 73 years recruited from 22 sites across the UK. This study included 502,611 participants with baseline measures collected between 2006 and 2010 [19], and data have since been linked to hospital and mortality records. A sub-sample of 103,704 also completed objective measures of PA between 2013 and 2015 [20]. Participants gave written informed consent prior to data collection, and ethical approval was obtained by the North-West Research Ethics Committee [21]. To minimise reverse causality (i.e. undiagnosed, subclinical disease(s) leading to lower exercise and mortality), we excluded participants who died within 2 years follow-up from baseline assessment (*n* = 2416) and also excluded participants with no data for the self-reported PA (*n* = 8256), leaving 491,939 participants for analysis.

### Defining multimorbidity

The UK Biobank collected self-reported medical information based on physician diagnosis [22]. Three sources of selecting long-term cardiovascular, non-cardiovascular, or mental health conditions were used [23]. The first included conditions from the quality and outcomes framework (QoF) which report the most common diseases in the UK [24]; the second was from a large UK-based study, containing 40 of the recommended core disorders for any multimorbidity measure [7]; and the last was from a systematic review on multimorbidity indices that included 17 conditions [25]. Based on these sources, we selected a total of 36 chronic conditions. Participants with two or more of these 36 chronic conditions were classified as having multimorbidity (Additional file 1: Methods S1).

### Mortality

Mortality data were obtained from the National Health Service (NHS) Information Centre for participants from England and Wales and the NHS Central Register for participants from Scotland. Data for survivors were censored on 31 January 2016 for England and Wales and 30 November 2015 for Scotland.

### Physical activity

PA was measured both subjectively and objectively. Two questionnaires were used for subjectively reported (self-reported) PA: (1) leisure-time PA (LTPA), which included five activities undertaken in the last 4 weeks—walking, light DIY (do-it-yourself), heavy DIY, strenuous sports, and other exercises, and (2) a modified version of the International Physical Activity Questionnaire, which assessed total physical activity (TPA) [26], including walking, moderate, and vigorous PA performed over the last 7 days. We categorised participants by three mutually exclusive groups: low (< 600 metabolic equivalent (MET)-min/week), moderate (600 to < 3000 MET-min/week), and high (≥ 3000 MET-min/week) PA based on a standard scoring criteria [26]; the threshold at 600 MET-min/week is equivalent to reaching the recommended guidelines (150 min per week) for moderate-intensity PA. Objective PA was measured using the Axivity AX3 wrist-worn triaxial accelerometer (Axivity Ltd., Newcastle, UK), where participants were requested to wear the monitor continuously for seven consecutive days [20]. We excluded participants with less than 3 days of wear data, without wear data in each 1-h period of the 24-h cycle, or with failed accelerometer calibration. We used the overall acceleration average, since it measured the total time spent across all levels of PA intensity. Within the population distribution, we categorised the overall acceleration average by tertiles and based the estimates on the median value. For ease of interpretation, based on the results of a laboratory calibration study [27], we described PA of each tertile in terms of walking ‘at a brisk pace for exercise’ of low (4 min/day), moderate (10 min/day), and high (22 min/day) (Additional file 1: Methods S2).

### Other covariates

Other relevant covariates, including sociodemographic (age, sex, ethnicity, education level, employment status, and socioeconomic status (Townsend deprivation index)) and lifestyle (smoking status, body mass index, alcohol consumption, fruit and vegetable portions, oily fish, non-oily fish, processed meat, red meat intake, and sedentary behaviour) factors, were also extracted (Additional file 1: Methods S3).

### Statistical analysis

We estimated the prevalence of multimorbidity, with the pattern of comorbidities illustrated using heat maps. Baseline characteristics stratified by multimorbidity status were summarised using numbers, proportions, and medians with interquartile range (IQR). We created an additional category for each sociodemographic and lifestyle covariate to account for missing data. For each PA measure, participants with complete PA data were included in the analyses, i.e. complete-case analysis for LTPA (*n* = 488,574), TPA (*n* = 454,639), and objective PA (*n* = 95,616). Spearman’s rank correlation was used to assess the agreement between the two continuous subjective PA measures and between the continuous subjective and objective PA measures. We used Cox proportional hazards regression, with time since baseline assessment as the start of follow-up, to model the association between PA and all-cause mortality in individuals with and without multimorbidity. Hazard ratios (HR) and corresponding 95% confidence intervals (95% CI) were calculated, and Schoenfeld’s residuals were used to verify the proportional hazards assumption. Four incremental models were fitted: model 1—unadjusted; model 2—adjusted for age and sex; model 3—additionally adjusted for sociodemographic factors; and model 4—additionally adjusted for lifestyle factors. The lowest PA group was the reference category. The calculation of years of life lost (i.e. difference in life expectancy) involved a two-step process using flexible parametric survival models with age as time scale. First, residual life expectancy was estimated as the area under the survival curve up to 100 years old, conditional on surviving at ages 45 to 100 years old (1-year intervals). Second, the differences in years of life were calculated as the difference between the areas under two survival curves [28], i.e. the difference between life expectancy for the moderate or high PA group with the reference of the low PA group by multimorbidity status. To calculate life expectancy, proportional hazard survival analyses were conducted with the stpm2 command which uses restricted cubic splines to model the baseline cumulative hazard [29, 30]. Statistical codes are available at *GitHub yc244*.

### Sensitivity analysis

We grouped self-reported PA measures in tertiles and calculated HR (95% CI) and differences in residual life expectancy. Additionally, the PA measures were investigated as continuous variables using a log base of 1.1 (i.e. log 1.1 (total PA)) to represent a 10% increase in total PA. Due to the time difference between the baseline and objective PA measurements, we further analysed the association between objective PA and mortality taking the time at objective PA assessment as the start of the follow-up. Model 1 was unadjusted, model 2 was adjusted for age at objective PA measurement and sex, and model 3 was further adjusted for ethnicity and socioeconomic status. Adjustments for other lifestyle factors were not possible as they may have changed between the baseline measurement and objective PA measurement. We also recalculated the HR and corresponding 95% CI for the association between objective PA and mortality; instead of the tertiles being based on the overall acceleration, we based the tertiles on the time accumulated above 250 m*g* of total acceleration (Additional file 1: Methods S2)**.** Since we categorised multimorbidity as any two or more chronic condition combinations, it is possible that combinations can include two “high-impact” (e.g. diabetes and heart failure) or “low-impact” (e.g. hypertension and asthma) conditions. Therefore, we performed two additional analyses to account for the severity of the conditions. Firstly, we used participants’ self-reported overall health rating (UK Biobank Data-Field 2178), as a proxy of severity, and categorised participants into four groups: good health without multimorbidity (reference), poor health without multimorbidity, good health with multimorbidity, and poor health with multimorbidity; participants with missing health rating were excluded from analyses (*n* = 2324). Secondly, we used the 10 most common comorbidities [31] and categorised participants into two groups: with and without top-10 comorbidity. We repeated the analyses using the three PA exposures. A table summarises main and sensitivity analyses (Additional file 1: Methods S5). All analyses were performed using Stata 14 MP.

## Results

### Participant characteristics

**Fig. 1:**
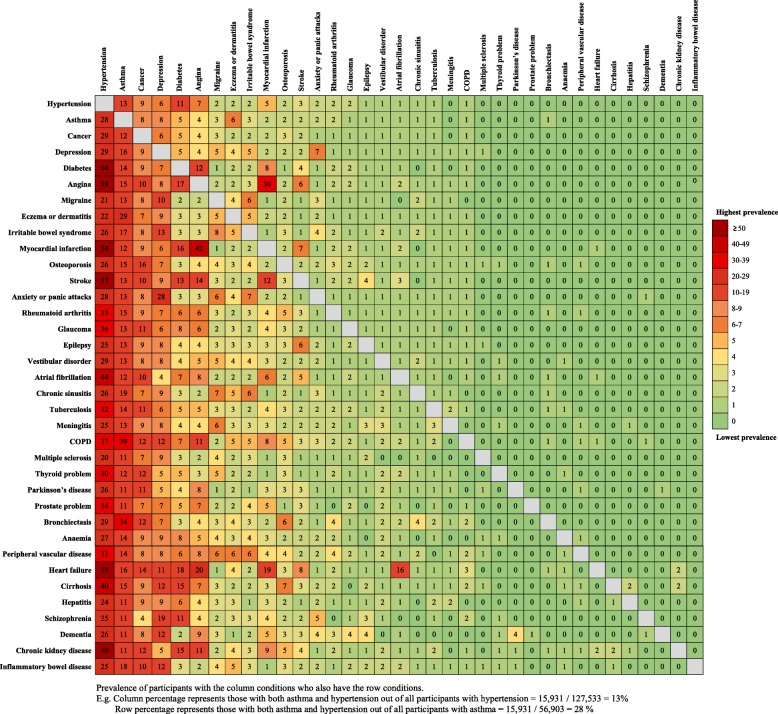
Comorbidity patterns of the included 36 chronic conditions (%) (*n* = 491,939)

The five most prevalent chronic conditions in 491,939 participants were hypertension (25.9%), asthma (11.6%), cancer (8.2%), depression (5.6%), and diabetes (4.2%). The most prevalent comorbidities include chronic kidney disease and hypertension (70%), diabetes and hypertension (64%), and myocardial infarction and angina (42%) (Fig. 1). The total number of conditions ranged from 0 to 10, where the overall prevalence of multimorbidity (≥ 2 chronic conditions) was 19.6% (*n* = 96,622). Most participants were white (94.3%), with a median (IQR) age of 58 years (50–63). Participants with multimorbidity were mainly older compared to participants without (> 60 years: 52% vs. 35%, respectively), lived in deprived areas (most deprived quintile: 24% vs. 19%, respectively), were overweight or obese (77% vs. 64%, respectively), had slightly lower consumption of alcohol (33% vs. 38% consuming ≥ 14 units/week, respectively), and spent more time in sedentary behaviour (26% vs. 20% spending ≥ 6 h, respectively) (Table 1). The two self-reported PA exposures were positively correlated (Pearson’s *r* = 0.41; *P* < 0.001) and between both subjective and objective PA measures (Pearson’s *r* = 0.19; *P* < 0.001). When categorised into low, moderate, and high PA levels, more than double of the participants with multimorbidity were in the low PA group than in the high PA group (objective PA 47% vs. 22%, respectively). We found similar baseline characteristics when accounting for the severity of the conditions, top-10 comorbidity, and the subsample of participants who undertook the objective PA. Participants who completed the objective PA measurements were generally from higher affluent areas, more educated, in employment, and less likely to be current smokers or obese compared to non-participants **(**Additional file 2: Tables S1, S2, S3, S4, S5, S6, S7, and S8, Figure S1).

**Table 1:** Participant characteristics by multimorbidity status (2 or more conditions)

Characteristics	All participants (*N* = 491,939)	Multimorbidity status		
		With multimorbidity (*n* = 96,622)	Without multimorbidity (*n* = 395,317)	*P* value
Sex				
Female	267,883 (54.5)	51,801 (53.6)	216,082 (54.7)	
Male	224,056 (45.5)	44,821 (46.4)	179,235 (45.3)	< 0.001
Age, years (median [IQR])	58 [50–63]	61 [54–65]	57 [49–63]	< 0.001
Age categories				
≤ 50	129,832 (26.4)	14,787 (15.3)	115,045 (29.1)	
51–60	173,712 (35.3)	31,659 (32.8)	142,053 (35.9)	
> 60	188,395 (38.3)	50,176 (51.9)	138,219 (35.0)	< 0.001
Ethnicity				
White	463,804 (94.3)	91,013 (94.2)	372,791 (94.3)	
Non-white	26,401 (5.4)	5222 (5.4)	21,179 (5.4)	
Missing	1734 (0.4)	387 (0.4)	1347 (0.3)	0.016
Socioeconomic status				
1st quintile (least deprived)	99,178 (20.2)	17,163 (17.8)	82,015 (20.7)	
2nd quintile	98,418 (20.0)	17,753 (18.4)	80,665 (20.4)	
3rd quintile	98,401 (20.0)	18,392 (19.0)	80,009 (20.2)	
4th quintile	98,325 (20.0)	19,647 (20.3)	78,678 (19.9)	
5th quintile (most deprived)	97,001 (19.7)	23,543 (24.4)	73,458 (18.6)	
Missing	616 (0.1)	124 (0.1)	492 (0.1)	< 0.001
Education level				
College or university degree	160,218 (32.6)	25,630 (26.5)	134,588 (34.0)	
A/AS level or equivalent	54,924 (11.2)	9791 (10.1)	45,133 (11.4)	
O levels/GCSEs or equivalent	130,813 (26.6)	24,154 (25.0)	106,659 (27.0)	
Other (e.g. NVQ, nursing, missing)	145,984 (29.7)	37,047 (38.3)	108,937 (27.6)	< 0.001
Employment status				
Working	283,151 (57.6)	39,205 (40.6)	243,946 (61.7)	
Retired	163,238 (33.2)	43,889 (45.4)	119,349 (30.2)	
Unemployed	8112 (1.6)	1635 (1.7)	6477 (1.6)	
Other (student, volunteer/missing)	37,438 (7.6)	11,893 (12.3)	25,545 (6.5)	< 0.001
BMI categories, kg/m^2^				
Underweight (< 18.5)	2515 (0.5)	431 (0.4)	2084 (0.5)	
Normal weight (18.5–24.9)	159,596 (32.4)	21,591 (22.3)	138,008 (34.9)	
Overweight (25.0–29.9)	208,375 (42.4)	38,546 (39.9)	169,829 (43.0)	
Obese (≥ 30.0)	119,081 (24.2)	35,450 (36.7)	83,631 (21.2)	
Missing	2372 (0.5)	604 (0.6)	1768 (0.4)	< 0.001
Smoking status				
Never	269,050 (54.7)	46,543 (48.2)	222,507 (56.3)	
Former	169,693 (34.5)	39,068 (40.4)	130,625 (33.0)	
Current	51,342 (10.4)	10,524 (10.9)	40,818 (10.3)	
Missing	1854 (0.4)	487 (0.5)	1367 (0.3)	< 0.001
Alcohol consumption				
Never or < 14 units/week	308,029 (62.6)	64,377 (66.6)	243,652 (61.6)	
Excess ≥ 14 units/week	183,249 (37.3)	32,088 (33.2)	151,161 (38.2)	
Missing	661 (0.1)	157 (0.2)	504 (0.1)	< 0.001
Meet fruit/vegetable guidelines				
No	304,709 (61.9)	58,682 (60.7)	246,027 (62.2)	
Yes	186,628 (37.9)	37,818 (39.1)	148,810 (37.6)	
Missing	602 (0.1)	122 (0.1)	480 (0.1)	< 0.001
Oily fish (≥ 1/week)				
No	218,092 (44.3)	40,954 (42.4)	177,138 (44.8)	
Yes	273,627 (55.6)	55,636 (57.6)	217,981 (55.1)	
Missing	230 (0.0)	32 (0.0)	198 (0.1)	< 0.001
Non-oily fish (≥ 1/week)				
No	167,300 (34.0)	31,915 (33.0)	135,385 (34.2)	
Yes	324,435 (66.0)	64,682 (66.9)	259,753 (65.7)	
Missing	204 (0.0)	25 (0.0)	179 (0.0)	< 0.001
Processed meat (≥ 2/week)				
No	339,723 (69.1)	65,198 (67.5)	274,525 (69.4)	
Yes	152,022 (30.9)	31,394 (32.5)	120,628 (30.5)	
Missing	194 (0.0)	30 (0.0)	164 (0.0)	< 0.001
Red meat (≥ 2/week)				
No	418,282 (85.0)	81,011 (83.8)	337,271 (85.3)	
Yes	73,542 (14.9)	15,598 (16.1)	57,944 (14.7)	
Missing	115 (0.0)	13 (0.0)	102 (0.0)	< 0.001
Sedentary behaviour, hours				
Low (< 4)	222,081 (45.1)	38,314 (39.7)	183,767 (46.5)	
Moderate (4–6)	166,435 (33.8)	33,326 (34.5)	133,109 (33.7)	
High (≥ 6)	102,911 (20.9)	24,839 (25.7)	78,072 (19.7)	
Missing	512 (0.1)	143 (0.1)	369 (0.1)	< 0.001
LTPA, MET-min/week, (median [IQR])	619 [200–1353]	464 [118–1154]	656 [228–1406]	< 0.001
LTPA categories, MET-min/week				
Low (< 600)	238,971 (48.6)	53,658 (55.5)	185,313 (46.9)	
Moderate (600 to < 3000)	215,517 (43.8)	36,760 (38.1)	178,757 (45.2)	
High (≥ 3000)	34,086 (6.9)	5199 (5.4)	28,887 (7.3)	
Missing	3365 (0.7)	1005 (1.0)	2360 (0.6)	< 0.001
TPA MET-min/week, (median [IQR])	1655 [737–3360]	1440 [636–3093]	1706 [780–3430]	< 0.001
TPA categories, MET-min/week				
Low (< 600)	90,698 (18.4)	20,823 (21.6)	69,875 (17.7)	
Moderate (600 to < 3000)	232,856 (47.3)	43,120 (44.6)	189,736 (48.0)	
High (≥ 3000)	131,085 (26.7)	22,655 (23.5)	108,430 (27.4)	
Missing	37,300 (7.6)	10,024 (10.4)	27,276 (6.9)	< 0.001
Objective m*g*, (median [IQR])^a^	27.1 [22.4–32.5]	24.5 [20.1–29.7]	27.6 [22.9–33.0]	< 0.001
Objective categories, min/day of brisk walking^a^				
Low (4)	31,919 (33.4)	7369 (47.2)	24,550 (30.7)	
Moderate (10)	31,871 (33.3)	4757 (30.5)	27,114 (33.9)	
High (22)	21,826 (33.3)	3481 (22.3)	28,345 (35.4)	< 0.001

Shown are numbers (%) unless stated otherwise. *P* value indicates the difference between categories (Chi-square test); continuous data (Wilcoxon rank-sum test). A/AS level or equivalent = higher school certificate; O levels/GCSEs = school certificate

*NVQ* National Vocational Qualification, *BMI* body mass index, *LTPA* leisure-time physical activity questionnaire, *TPA* total physical activity questionnaire, *MET* metabolic equivalent of task, *mg* milli-gravitational units

^a^Data taken at the time of objective physical activity measurements

### Subjective physical activity and all-cause mortality

**Fig. 2:**
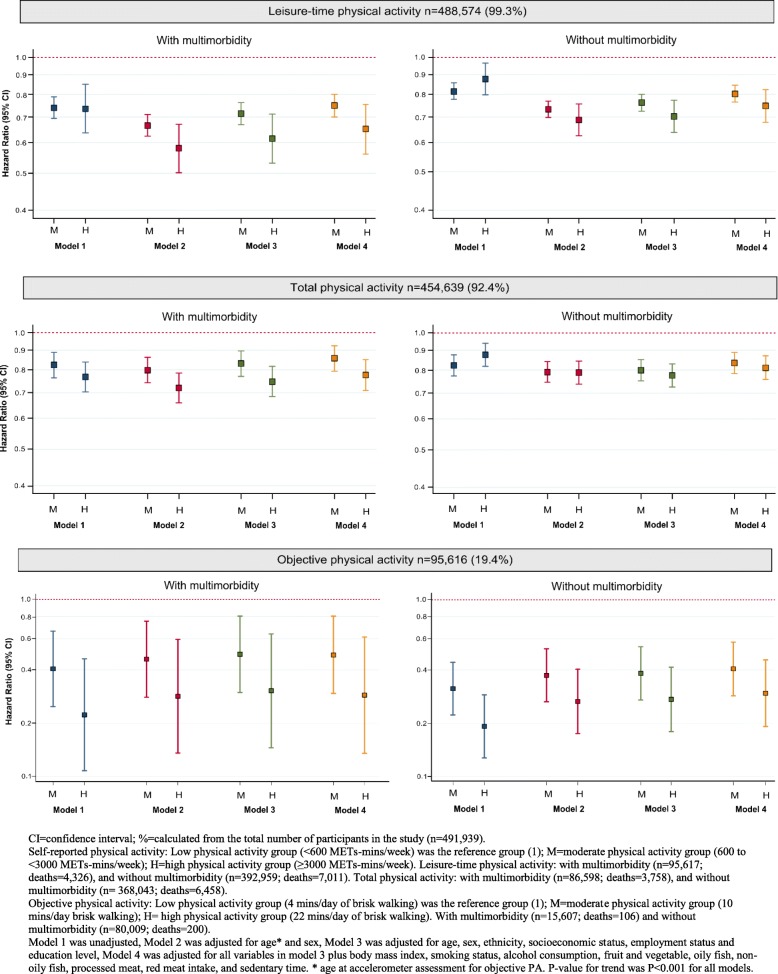
Association between moderate and high physical activity (PA) and mortality when compared to the low PA group for participants with and without multimorbidity (2 or more conditions)

The median follow-up was 7.0 [range 2.0–9.1] years with 11,479 recorded deaths, of which 38% (*n* = 4399) occurred in participants with multimorbidity. Self-reported PA showed an inverse dose-response association with mortality for all participants, independent of multimorbidity status and adjustments for confounders (Fig. 2). Using LTPA, in participants with multimorbidity, the risk of mortality was 25% lower in the moderate PA group (HR 0.75, 95% CI 0.70–0.80) and 35% lower in the high PA group (HR 0.65, 95% CI 0.56–0.75), compared to the low PA group in the fully adjusted model; corresponding figures for participants without multimorbidity were 20% (HR 0.80, 95% CI 0.76–0.85) and 25% (HR 0.75, 95% CI 0.68–0.82), respectively. Using TPA, in participants with multimorbidity, the risk of mortality was 14% lower in the moderate PA group (HR 0.86, 95% CI 0.79–0.93) and 22% lower in the high PA group (HR 0.78, 95% CI 0.71–0.85), compared to the low PA group, in the fully adjusted model; corresponding figures for participants without multimorbidity were 17% (HR 0.83, 95% CI 0.78–0.89) and 19% (HR 0.81, 95% CI 0.76–0.87), respectively. Results were consistent in sensitivity analyses with (1) LTPA and TPA categorised into tertiles, (2) continuously measured (log-transformed) PA, (3) alternative definitions of multimorbidity, accounting for overall health rating, or top-10 comorbidity (Additional file 2: Tables S10, S11 and 12, Figure S2).

### Objective physical activity and all-cause mortality

**Fig. 3:**
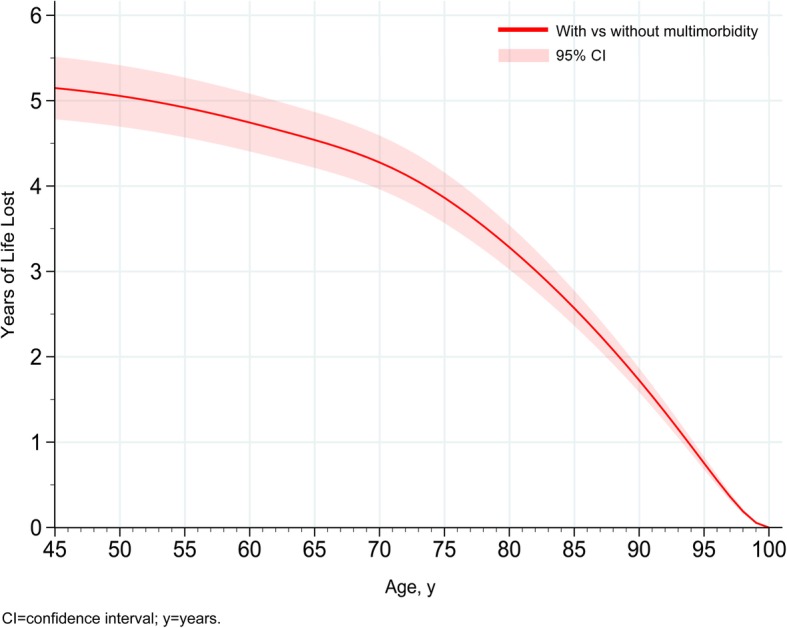
Modelling years of life lost by multimorbidity (2 or more conditions)

95,616 participants completed the objective PA measurements, of which 16.3% (*n* = 15,607) participants had multimorbidity. Using time since baseline assessment as the start of the follow-up, the median follow-up was 6.9 (range 3.8–9.1) years with 306 participant (0.3%) deaths. The amount of time spent walking ‘at a brisk pace for exercise’ (> 250 m*g*) was 4, 10, and 22 min/day in the low, moderate, and high volume PA groups. In the fully adjusted model, the risk of mortality in participants with multimorbidity was 51% lower in the moderate PA group (HR 0.49, 95% CI 0.29–0.80) and 71% lower in the high PA group, (HR 0.29, 95% CI 0.13–0.61) compared to the low PA group (Fig. 3). In participants without multimorbidity, there was a 60% (HR 0.40, 95% CI 0.29–0.57) and 71% (HR 0.29, 95% CI 0.19–0.46) lower risk of mortality in the moderate and high PA group respectively, compared to low PA (Fig. 2). Results were consistent in sensitivity analyses considering the following: (1) the objective PA assessment as the start of the follow-up (median follow-up was 1.23 (IQR 0.70, 1.72) years), (2) tertiles of time accumulated above 250 m*g* of total acceleration, (3) log-transformed objective PA, (4) alternative definitions of multimorbidity, accounting for overall health rating, or top-10 conditions (see Additional file 2: Tables S10, S11, S12 and S13, Figure S3).

### Life expectancy

#### Multimorbidity

At the age of 45 years, participants with multimorbidity had, on average, a life expectancy which was 5.15 (95% CI 4.78, 5.14) lower than participants without multimorbidity; the corresponding figure at the age of 65 years was 4.54 (95% CI 4.21, 4.87) years (Fig. 3). In sensitivity analyses, when accounting for the severity of the conditions, at 45 years, participants with poor health and multimorbidity had 9.00 (95% CI 8.47, 9.53) years lower life expectancy compared to those with good health without multimorbidity, while participants with top-10 comorbidity had 6.53 (95% CI 6.01, 7.05) years lower life expectancy compared to those without.

#### Self-reported physical activity

**Fig. 4:**
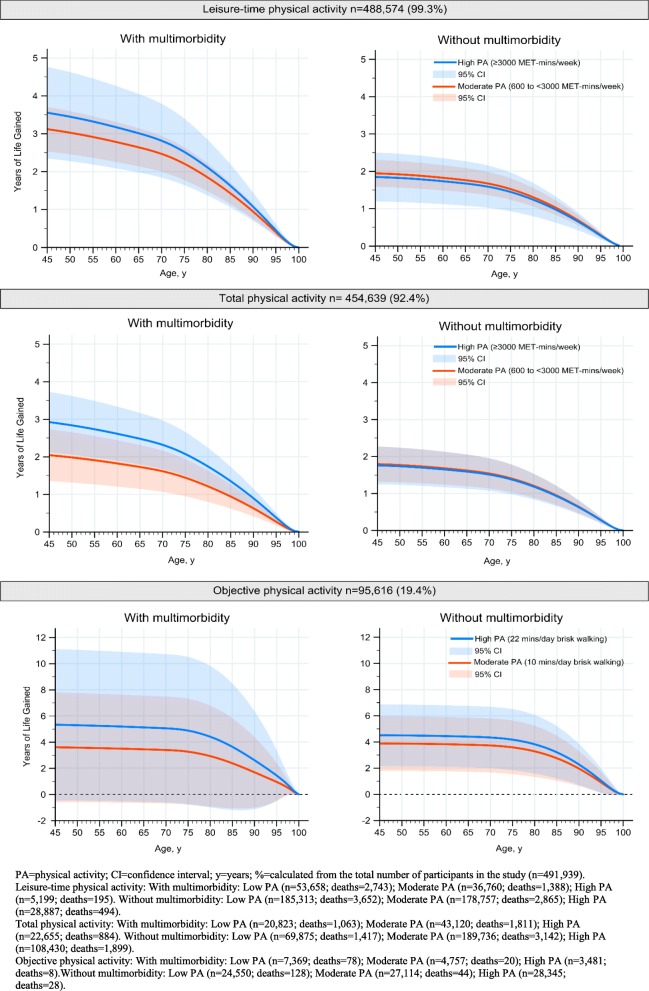
Life year gains associated with moderate and high physical activity (PA) when compared to the low PA group for participants with and without multimorbidity (2 or more conditions)

Using LTPA, in participants aged 45 years with multimorbidity, moderate and high PA were associated with an average of 3.12 (95% CI 2.53, 3.71) and 3.55 (95% CI 2.34, 4.77) additional life years gained, respectively, compared to low PA; corresponding figures in those without multimorbidity were 1.95 (95% CI 1.59, 2.31) and 1.85 (95% CI 1.19, 2.50) additional life years gained, respectively (Fig. 4). The effects of increased PA were lower in older participants such that the benefit was found to decrease with age. Using TPA, in participants aged 45 years with multimorbidity, moderate and high PA were associated with 2.04 (95% CI 1.35, 2.74) and 2.92 (95% CI 2.12, 3.73) additional life years gained, respectively, compared to low PA; corresponding figures in those without multimorbidity in the same group were 1.79 (95% CI 1.32, 2.27) and 1.76 (95% CI 1.25, 2.27) additional life years, respectively (Fig. 4). Results were similar in sensitivity analyses using tertiles rather than PA categories, considering the severity of the conditions or top-10 comorbidity (Additional file 2: Tables S9, S11, and S12).

#### Objective physical activity

Using objective PA, in participants with multimorbidity aged 45 years, moderate and high PA groups were associated with 3.60 (95% CI − 0.60, 7.79) and 5.32 (95% CI − 0.47, 11.11) additional life years gained, respectively, compared to the low PA group; corresponding values for those without multimorbidity were 3.88 (95% CI 1.79, 6.00) and 4.51 (95% CI 2.15, 6.88) additional life years, respectively (Fig. 4). In sensitivity analyses, results were comparable when accounting for the severity of the conditions or top-10 comorbidity (Additional file 2: Tables S11 and S12).

## Discussion

We found an inverse dose-response association between PA and mortality in participants with and without multimorbidity, which persisted even after adjustments for sociodemographic and lifestyle factors. For self-reported PA, achieving volumes of PA that are consistent with the current guidelines of 150 min of moderate-intensity PA per week was associated with a longer life expectancy, with little additional benefit seen above this amount. Moreover, results for objectively measured PA suggested as little as 10 min of brisk walking a day was associated with a longer life expectancy, again with little additional benefit seen at higher categories.

Only two studies have assessed the relationship between self-reported PA and mortality in people with multimorbidity. The first study from Spain (*n* = 3967), using LTPA, showed that in participants with multimorbidity (*n* = 571), being physically active was related to a 35% (95% CI 16–50%) lower risk of mortality, compared to being inactive [13]. Our results using LTPA mirror these findings, whereby there was a 35% lower risk in the high PA group compared to the low PA group. In contrast, results from the analysis of the National Health and Nutrition Examination Survey dataset (*n* = 16,091) found that LTPA had a minimal effect on the multimorbidity-mortality relationship. The models were stratified by PA levels, and the number of chronic conditions was the exposure variable. Results showed that for each additional chronic condition, the association with mortality remained strongly significant, regardless of the amount of PA (i.e. stratifying by ≥ 4000 MVPA MET-min/month indicated for each additional chronic condition, there was a 22% higher risk of mortality). However, for those who performed ≥ 8000 MET-min/month, the multimorbidity-mortality relationship was much smaller (HR 1.08, 95% CI 0.87–1.33), suggesting that people with multimorbidity would gain a benefit only if they report extremely high levels of PA [14]. Other studies in large general populations support our findings, as they show that both moderate and vigorous-intensity activities were associated with a longevity benefit [32–34]. Although there are no studies that examine the comparative role of PA and multimorbidity on life expectancy, our results are in line with previous research based on different populations [12, 34–36].

This study has some limitations. Firstly, participation in the UK Biobank was voluntary with slightly higher representation from affluent groups; therefore, the results may not be completely representative of the UK population [37]. The sub-sample who undertook the objective PA measurements was relatively healthier than those who did not participate and was limited to only computer literate participants [38]; this limits the representativeness of the sample for objective PA analysis, and these results should be interpreted with caution. Another limitation is related to the time difference between the baseline assessment and objective PA assessment: we analysed and adjusted for covariates at baseline (i.e. shifting the objective PA backwards), thus assuming the PA levels of the participants did not change between baseline and objective PA assessment, which may not be true. Nevertheless, objective PA measures have been used in a similar fashion in another UK Biobank analysis [39]. Furthermore, data from a longitudinal study on older adults in England reported steady PA levels over time with a slight decline in the time spent in vigorous-intensity activities [40]. Therefore, our results may have slightly under-estimated the association between objective PA and mortality. However, the hazard ratio results remained unchanged when we used the time of objective PA assessment as the start of the follow-up. Moreover, the short follow-up for the cohort of participants with objective PA did not allow a landmark analysis excluding participants who died in the first 2 years. There is currently no standard method for measuring multimorbidity [41]; however, our definition included most of the core conditions for any multimorbidity measure [7, 23, 25], and we have carried out sensitivity analyses using two different methods to ensure our findings are valid. Since this study was observational, we cannot derive any causality from the relationships we evidenced, yet there is substantial evidence supporting a causal relationship between higher levels of PA and good health status [10, 11, 18, 34, 36]. Finally, although we found an association between PA and higher life expectancy, especially in people with multimorbidity, we were unable to assess whether this translates to a better quality of life.

Strengths of this study include the large sample size used to assess the association between PA with mortality and life expectancy by multimorbidity status. Also, this is the first study to use objective PA to assess its association with mortality in people with multimorbidity. We controlled for a wide range of important confounders, including age, as this is related to both PA and multimorbidity; however, we cannot completely exclude the potential for residual confounding. We also removed deaths occurring within the first 2 years at baseline to minimise the risk of reverse causality. We used three different measures of PA and three different multimorbidity definitions to ensure that the findings are robust. When accounting for the severity of the conditions, we found that the message was even stronger for those with a poor health rating and multimorbidity, or with top-10 comorbidity, because the benefits of physical activity had a much greater impact on the years of life gained compared to those who had a good health state or without top-10 comorbidity.

## Conclusions

In conclusion, our findings showed an inverse dose-response association between PA and all-cause mortality suggesting the mortality benefits of PA still apply in people with multimorbidity. Moreover, our results indicated that self-reported levels of physical activity that are consistent with the current physical activity guidelines, or as little as 10 min of brisk walking a day based on objective measurement, were associated with longer life expectancy with little additional benefit observed above these levels, implying that it is not necessary to engage in high-volume or high-intensity PA to achieve the potential health benefits. However, we identified a large number of participants with multimorbidity who did not reach this level of PA, suggesting that there is a need for individuals with multimorbidity to increase their PA in ways that are achievable.

## Additional files

Additional file 1: Methods S1 List of the 36 chronic conditions included within the definition of multimorbidity. Methods S2 Physical activity measurements. Methods S3 Additional methods for sociodemographic and lifestyle factors. Methods S4 Summary of main and sensitivity analyses. (DOCX 31 kb)

Additional file 2 **Table S1.** Number of participants with comorbidity. **Table S2.** Number of participants by the total number of chronic conditions**.** **Table S3.** Most to least prevalent chronic conditions by overall health rating and multimorbidity status. **Table S4.** Participant characteristics by overall health status and multimorbidity. **Table S5.** Number of participants with the top-10 comorbidity. **Table S6.** Characteristics by participants with and without top-10 comorbidity. **Table S7.** Participant characteristics who undertook the objective physical activity by multimorbidity status. **Table S8.** Participants characteristics by completion of the objective physical activity measurements. **Table S9.** Self-reported physical activity in tertiles and years of life gained at the age of 45 years by multimorbidity status. **Table S10.** Association between continuously measured (log-transformed) physical activity and mortality. **Table S11.** Association between physical activity and mortality for participants with good or poor overall health rating and multimorbidity status, and years of life gained at the age of 45 years. **Table S12.** Association between physical activity and mortality for participants with and without top-10 comorbidity, and years of life gained at the age of 45 years. **Table S13.** Association between objective physical activity in tertiles of time accumulated above 250 m*g* of total acceleration and mortality. **Figure S1.** Most to least prevalent chronic conditions used to define multimorbidity (*n* = 491,939). **Figure S2.** Association between self-reported physical activity in tertiles and mortality. **Figure S3.** Association between objective physical activity and mortality, taking the time at objective PA assessment as the start of the follow-up in participants with and without multimorbidity. (DOCX 2092 kb)

## Abbreviations

A/AS Level: Advanced level qualifications
BMI: Body mass index
CI: Confidence intervals
DIY: Do-it-yourself
GCSE: General certificate of secondary education
HR: Hazard ratio
IPAQ: International Physical Activity Questionnaire
IQR: Interquartile range
Log: Logarithm
LTPA: Leisure-time physical activity
MET: Metabolic equivalent
m*g*: Milli-gravitational units
MP: Multicore processor
MVPA: Moderate to vigorous physical activity
NHS: National Health Service
NVQ: National Vocational Qualification
PA: Physical activity
QoF: Quality and outcomes framework
TPA: Total physical activity
UK: United Kingdom
