# Supplementary material for: Physical activity, multimorbidity, and life expectancy: a UK Biobank longitudinal study
Source: BMC Med. 2019 Jun 12;17:108. doi: 10.1186/s12916-019-1339-0 (PMC6560907; doi:10.1186/s12916-019-1339-0)
Supplement: Supplementary file 1 — Methods S1 List of the 36 chronic conditions included within the definition of multimorbidity. Methods S2 Physical activity measurements. Methods S3 Additional methods for sociodemographic and lifestyle factors. Methods S4 Summary of main and sensitivity analyses. (DOCX 31 kb) [file 12916_2019_1339_MOESM1_ESM.docx]

**Additional File 1 - Supplementary Methods**

# Methods S1. List of the 36 chronic conditions included within the definition of multimorbidity

| - Anaemia |
| --- |
| - Angina |
| - Anxiety or panic attacks |
| - Asthma |
| - Atrial fibrillation |
| - Bronchiectasis |
| - Cancer |
| - Chronic kidney disease |
| - Chronic obstructive pulmonary disease |
| - Chronic sinusitis |
| - Cirrhosis |
| - Dementia |
| - Depression |
| - Diabetes |
| - Eczema or dermatitis |
| - Epilepsy |
| - Glaucoma |
| - Heart failure |
| - Hepatitis |
| - Hypertension |
| - Inflammatory bowel disease |
| - Irritable bowel syndrome |
| - Meningitis |
| - Migraine |
| - Multiple sclerosis |
| - Myocardial infarction |
| - Osteoporosis |
| - Parkinson’s disease |
| - Peripheral vascular disease |
| - Prostate problem |
| - Rheumatoid arthritis |
| - Schizophrenia |
| - Stroke |
| - Thyroid problem |
| - Tuberculosis |
| - Vestibular disorder |

# Methods S2. Physical activity measurements

**Subjective physical activity**

Leisure-time physical activity (LTPA) included five activities undertaken in the last four weeks: walking for pleasure, light DIY (do-it-yourself), heavy DIY, strenuous sports, and other exercises. Participants were asked to quantify the frequency and duration of their participation, which was converted to an average weekly value. A metabolic equivalent (MET) value was assigned to walking for pleasure (3.5), light DIY (1.5), heavy DIY (5.5), strenuous sports (8.0) other exercises (4.0), and none of the five activities (0).[1] Similarly, total physical activity (TPA) which was a modified version of the International Physical Activity Questionnaire (IPAQ),[2] was assessed through capturing the frequency and duration of walking, moderate, and vigorous PA performed over the last seven days. Data were analysed in accordance with the IPAQ scoring protocol.[2] MET levels included walking (3.3), moderate-intensity (4.0), and vigorous-intensity (8.0).[1] For both the measures, total weekly PA (MET-mins/week) was calculated by multiplying the frequency, duration, and the MET values.[1] Based on the standard scoring criteria of PA, we grouped the sample as low PA (<600 MET-mins/week), moderate PA (600 to <3000 MET-mins/week), and high PA (≥3000 MET-mins/week);[2] the threshold at 600 MET-mins/week is equivalent to reaching the recommended guidelines (150 minutes per week) for moderate-intensity PA.

**Objective physical activity**

Objective PA was assessed using the Axivity AX3 wrist-worn triaxial accelerometer (Axivity Ltd, Newcastle, UK), where participants were requested to wear the monitor continuously for seven consecutive days.[3] Participants who provided a valid email address at the baseline assessment were re-contacted and invited to wear an accelerometer. The accelerometer was posted to their home address following consent. Full data processing methods are available elsewhere.[4] We excluded participants with less than three days of wear data and without wear data in each one-hour period of the 24-hour cycle (n=6,943) as recommended, [4] or with failed accelerometer calibration (n=162); or for participants with objective PA measurements after the mortality register was taken (n=121). We used the overall acceleration average, since this measured the total time spent in different levels of PA intensity and has previously demonstrated a high correlation with total energy expenditure.[5] Within the population distribution, we established tertiles to group PA: lowest (<24.0 milli-gravitational units (m*g*)), middle (24.0-30.4 m*g*) and upper tertile (>30.4 m*g*). For ease of interpretation, we described the tertiles in terms of the number of minutes accumulated at an intensity equivalent to, or greater than, walking ‘at a brisk pace, for exercise’ (4.3 MET),[1] which can be predicted from the time accumulated at an acceleration above 250 m*g*. [6] The 250 m*g* cut-point is taken from data from a laboratory-based calibration study. [6] This study included 30 adults aged 18-65 years and the following activities: lying, sitting, standing, lifestyle activities circuit, slow walking, fast walking, running, walking up and down a step. We chose to translate activity levels in terms of brisk walking as it is a moderate intensity activity that is frequently cited as an example in physical activity guidelines (e.g. <https://www.nhs.uk/live-well/exercise/>). The energy expenditure compendium reports 4.3 MET is indicative of walking at a ‘brisk pace for exercise’. [1] The regression equation presented by the authors of the calibration study for wrist acceleration and energy expenditure in adults showed that an acceleration of 250 m*g* predicted 4.3 MET. We used the fraction time <= 250m*g* acceleration (Data-Field 90133) to carry out the calculation. Based on this, the median (interquartile range) value of the lowest tertile equated to 4.3 (2.9-7.2) mins/day of walking at brisk pace. Similarly, the middle tertile equated to 10.1 (7.2-14.4) mins/day; and upper tertile to 21.6 (15.8-30.2) mins/day of brisk walking. Therefore, for ease of interpretation, we categorised objective PA in terms of low PA (4 mins/day of brisk walking), moderate PA (10 mins/day of brisk walking), and high PA (22 mins/day of brisk walking). To ensure our results were valid, we further carried out sensitivity analyses where we based the tertiles only on the population who spent time above 250 m*g*. The median (interquartile range) value of the lowest tertile equated to 4.3 (2.9-5.8) mins/day, the middle tertile 11.5 (10.1-14.4) mins/day; and upper tertile 24.5 (20.2-31.7) mins/day of brisk walking.

**References**

1. Ainsworth BE, Haskell WL, Whitt MC, Irwin ML, Swartz AM, Strath SJ, O Brien WL, Bassett DR, Schmitz KH, Emplaincourt PO: **Compendium of physical activities: an update of activity codes and MET intensities**. *Medicine and science in sports and exercise* 2000, **32**(9; SUPP/1):S498-S504.

2. **IPAQ scoring protocol - International Physical Activity Questionnaire** [<https://sites.google.com/site/theipaq/scoring-protocol>]

3. **UK Biobank Physical activity monitor (accelerometer)** [<https://biobank.ctsu.ox.ac.uk/crystal/docs/PhysicalActivityMonitor.pdf>]

4. Doherty A, Jackson D, Hammerla N, Plötz T, Olivier P, Granat MH, White T, van Hees VT, Trenell MI, Owen CG: **Large scale population assessment of physical activity using wrist worn accelerometers: the UK Biobank study**. *PloS one* 2017, **12**(2):e0169649.

5. van Hees VT, Renström F, Wright A, Gradmark A, Catt M, Chen KY, Löf M, Bluck L, Pomeroy J, Wareham NJ: **Estimation of daily energy expenditure in pregnant and non-pregnant women using a wrist-worn tri-axial accelerometer**. *PloS one* 2011, **6**(7):e22922.

6. Hildebrand M VHV, Hansen BH, Ekelund U.: **Age-group comparibility of raw accelerometer output from wrist- and hip-worn monitors**. *Medicine and Science in Sports and Exercise* 2014, **46**(9):1816-1824.

#

# Methods S3. Additional methods for sociodemographic and lifestyle factors

**Smoking**

Participants were asked about their smoking status during the assessment; responses were categorised as current, former, never smoked, and missing.

**Body Mass Index**

The body mass index (BMI) value was constructed from the height (cm) and weight (kg) measured during the physical assessments (weight divided by the square of the height in metres). BMI was categorised as: underweight (<18.5 kg/m^2^), normal (between ≥18.5 and <25 kg/m^2^), overweight (between ≥25 and <30 kg/m^2^), obese (≥30 kg/m^2^), and missing.

**Diet**

Participant’s diet was assessed by their consumption of fruit, vegetable, meat, and fish. To calculate the fruit and vegetable intake, the combined responses for fresh fruit (pieces), dried fruit (pieces), salad/raw vegetable (heaped tablespoons), and cooked vegetable (heaped tablespoons), were converted into proportions and grouped to indicate whether participants ate <5 or ≥5 portions/day, based on the NHS guidelines,[1] or had missing information. Two types of fish were recorded, oily (e.g. sardines, salmon, mackerel, herring) and non-oily (e.g. cod, tinned tuna, haddock). Participants were asked how often these were consumed. The answers were categorised as less than once a week, more than once a week, or missing. Similarly, the overall red meat consumption consisted of pork, lamb and beef. The three meats were combined and categorised as less than two a week, more than two a week, or missing. Likewise, the processed meat intake was categorised as less than two a week, more than two a week, or missing.

**Alcohol**

The UK Biobank asked participants for the number of pints of beer, glasses of wine, and measures of spirit consumed in the last week. Alcoholic drinks differ in the amount of alcohol content, therefore each drink was converted into equivalent standard units, where 1 unit contained 10 ml of ethyl alcohol.[2] The guidelines from the Office for National Statistics (ONS) were used as this was the most updated method of converting volumes to units.[2] Total weekly units of alcohol were calculated by adding the units of beer, wine, and spirits. Excess alcohol consumption was defined as more than 14 units of alcohol a week based on the NHS guidelines.[3]

**Sedentary behaviour**

To measure the total sedentary time, the sum of self-reported hours spent watching television (TV), using the computer, and driving were derived on a typical day. Values greater than 24 hours per day were excluded, and those reporting over 16 hours were re-coded to 16 hours. Tertiles based on the sample population was used to categorise sedentary time into low (0 to 4 hours), medium (5 to 6 hours), and high (more than 6 hours) levels of sedentary behaviour.

**Education level**

Education level was categorised as having a College or University degree, A levels/AS levels or equivalent, O levels/GCSEs or equivalent, or other qualification (CSEs, NVQ, HND, HNC, professional qualifications (e.g. nursing, teaching) and if the information was missing). The first entered qualification was assessed as the highest qualification.

**Employment status**

Employment status was grouped as working (in paid employment or self-employed), unemployed, retired, or other (looking after home and/or family, unable to work because of sickness or disability, doing unpaid or voluntary work, full or part time student, or did not provide an answer). The first entered employment was assumed as their most current employment status.

**Socioeconomic status**

Townsend deprivation index was used as a measure of socioeconomic status. This measure combines census data on housing, employment, social class, and car availability based on the postal code of participants. The Townsend deprivation index has been validated for use in a UK-based population.[4] The index was categorised into quintiles from the sample population, with the least deprived (quintile 1) to the most deprived (quintile 5).

**References**

1. NHS, **5 A Day portion sizes,** Available at: [<http://www.nhs.uk/Livewell/5ADAY/Pages/Portionsizes.aspx>]

2. Goddard E: **Estimating alcohol consumption from survey data: updated method of converting volumes to units**: Office for National Statistics Newport; 2007.

3. NHS, **Alcohol units,** Available at: [<https://www.nhs.uk/Livewell/alcohol/Pages/alcohol-units.aspx>]

4. Townsend P: **Poverty in the United Kingdom** In*.* London: Allen Lane and Penguin Books; 1979.

# Methods S4. Summary of main and sensitivity analyses

✓ Main analyses;

X Sensitivity analyses;

– Analysis not performed

LTPA=Leisure-time physical activity; TPA=Total physical activity; PA=physical activity; HR=hazard ratio; LE=Life expectancy.

| **Multimorbidity**  **definition** | **Baseline characteristics** | **Pattern of comorbidity** | **Self-reported LTPA**  **(HR and LE)** | **Self-reported LTPA tertiles**  **(HR and LE)** | **Self-reported TPA**  **(HR and LE)** | **Self-reported TPA tertiles**  **(HR and LE)** | **Objective PA tertiles**  **(HR and LE)** | **All three PA continuous**  **(HR)** | **Objective PA tertiles >250m*g***  **(HR)** |
| --- | --- | --- | --- | --- | --- | --- | --- | --- | --- |
| **2 or more conditions** |  |  |  | **X** |  | **X** |  | **X** | **X** |
| **2 or more conditions combined with self-reported overall health** | **X** | **X** | **X** | – | **X** | – | **X** | – | – |
| **2 or more top-10 most common comorbidities** | **X** | **X** | **X** | – | **X** | – | **X** | – | – |
