# Supplementary material for: Physical activity, multimorbidity, and life expectancy: a UK Biobank longitudinal study
Source: BMC Med. 2019 Jun 12;17:108. doi: 10.1186/s12916-019-1339-0 (PMC6560907; doi:10.1186/s12916-019-1339-0)
Supplement: Supplementary file 2 — Table S1. Number of participants with comorbidity. Table S2. Number of participants by the total number of chronic conditions. Table S3. Most to least prevalent chronic conditions by overall health rating and multimorbidity status. Table S4. Participant characteristics by overall health status and multimorbidity. Table S5. Number of participants with the top-10 comorbidity. Table S6. Characteristics by participants with and without top-10 comorbidity. Table S7. Participant characteristics who undertook the objective physical activity by multimorbidity status. Table S8. Participants characteristics by completion of the objective physical activity measurements. Table S9. Self-reported physical activity in tertiles and years of life gained at the age of 45 years by multimorbidity status. Table S10. Association between continuously measured (log-transformed) physical activity and mortality. Table S11. Association between physical activity and mortality for participants with good or poor overall health rating and multimorbidity status, and years of life gained at the age of 45 years. Table S12. Association between physical activity and mortality for participants with and without top-10 comorbidity, and years of life gained at the age of 45 years. Table S13. Association between objective physical activity in tertiles of time accumulated above 250 mg of total acceleration and mortality. Figure S1. Most to least prevalent chronic conditions used to define multimorbidity (n = 491,939). Figure S2. Association between self-reported physical activity in tertiles and mortality. Figure S3. Association between objective physical activity and mortality, taking the time at objective PA assessment as the start of the follow-up in participants with and without multimorbidity. (DOCX 2092 kb) [file 12916_2019_1339_MOESM2_ESM.docx]

**Additional File 2: Supplementary Tables and Figures**

# Table S1. Number of participants with comorbidity

# Table S2. Number of participants by the total number of chronic conditions

| Total number of conditions | Frequency | % | Cumulative % |
| --- | --- | --- | --- |
| 0 | 229,071 | 46.56 | 46.56 |
| 1 | 166,246 | 33.79 | 80.36 |
| 2 | 67,739 | 13.77 | 94.13 |
| 3 | 21,052 | 4.28 | 98.41 |
| 4 | 5,788 | 1.18 | 99.58 |
| 5 | 1,553 | 0.32 | 99.90 |
| 6 | 360 | 0.07 | 99.97 |
| 7 | 108 | 0.02 | 100.00 |
| 8 | 20 | 0.00 | 100.00 |
| 9 | 1 | 0.00 | 100.00 |
| 10 | 1 | 0.00 | 100.00 |

# Table S3. Most to least prevalent chronic conditions by overall health rating and multimorbidity status

| Good health &  without multimorbidity  (n = 317,264) | | | Poor health &  without multimorbidity  (n = 76,495) | | | Good health &  with multimorbidity  (n = 48,588) | | | Poor health &  with multimorbidity  (n = 47,268) | | |
| --- | --- | --- | --- | --- | --- | --- | --- | --- | --- | --- | --- |
| 1 | **Hypertension** | 14.8 | **1** | **Hypertension** | 22.9 | **1** | **Hypertension** | 62.4 | **1** | **Hypertension** | 67.5 |
| 2 | **Asthma** | 6.3 | **2** | **Asthma** | 7.9 | **2** | **Asthma** | 32.4 | **2** | **Asthma** | 31.3 |
| 3 | **Cancer** | 4.6 | **3** | **Cancer** | 5.2 | **3** | **Cancer** | 24.9 | **3** | **Diabetes** | 22.3 |
| 4 | **Migraine** | 1.7 | **4** | **Depression** | 4.5 | **4** | **Depression** | 15.0 | **4** | **Depression** | 20.1 |
| 5 | **Eczema or dermatitis** | 1.2 | **5** | **Diabetes** | 2.3 | **5** | **Diabetes** | 12.4 | **5** | **Cancer** | 19.4 |
| 6 | **Irritable bowel syndrome** | 1.0 | **6** | **Irritable bowel syndrome** | 1.6 | **6** | **Eczema or dermatitis** | 10.0 | **6** | **Angina** | 18.2 |
| 7 | **Diabetes** | 0.7 | **7** | **Migraine** | 1.5 | **7** | **Migraine** | 9.4 | **7** | **Myocardial infarction** | 12.0 |
| 8 | **Osteoporosis** | 0.7 | **8** | **Angina** | 1.3 | **8** | **Angina** | 8.5 | **8** | **Irritable bowel syndrome** | 7.4 |
| 9 | **Angina** | 0.5 | **9** | **Rheumatoid arthritis** | 1.2 | **9** | **Irritable bowel syndrome** | 7.0 | **9** | **Stroke** | 6.8 |
| 10 | **Anxiety or panic attacks** | 0.5 | **10** | **Myocardial infarction** | 1.0 | **10** | **Myocardial infarction** | 6.4 | **10** | **Migraine** | 6.3 |
| 11 | **Glaucoma** | 0.5 | **11** | **Eczema or dermatitis** | 0.9 | **11** | **Osteoporosis** | 4.9 | **11** | **Eczema or dermatitis** | 5.9 |
| 12 | **Depression** | 0.4 | **12** | **Osteoporosis** | 0.9 | **12** | **Anxiety or panic attacks** | 4.6 | **12** | **Osteoporosis** | 5.1 |
| 13 | **Epilepsy** | 0.4 | **13** | **Anxiety or panic attacks** | 0.7 | **13** | **Glaucoma** | 3.8 | **13** | **Rheumatoid arthritis** | 5.0 |
| 14 | **Myocardial infarction** | 0.4 | **14** | **Epilepsy** | 0.6 | **14** | **Stroke** | 3.6 | **14** | **Anxiety or panic attacks** | 4.6 |
| 15 | **Vestibular disorder** | 0.4 | **15** | **Multiple sclerosis** | 0.6 | **15** | **Vestibular disorder** | 2.8 | **15** | **Glaucoma** | 3.0 |
| 16 | **Chronic sinusitis** | 0.3 | **16** | **Stroke** | 0.6 | **16** | **Chronic sinusitis** | 2.3 | **16** | **Atrial fibrillation** | 2.9 |
| 17 | **Rheumatoid arthritis** | 0.3 | **17** | **Atrial fibrillation** | 0.5 | **17** | **Atrial fibrillation** | 2.2 | **17** | **Epilepsy** | 2.6 |
| 18 | **Atrial fibrillation** | 0.2 | **18** | **Glaucoma** | 0.5 | **18** | **Rheumatoid arthritis** | 2.2 | **18** | **Vestibular disorder** | 2.3 |
| 19 | **Meningitis** | 0.2 | **19** | **Vestibular disorder** | 0.4 | **19** | **Epilepsy** | 2.1 | **19** | **Chronic obstructive pulmonary disease** | 2.1 |
| 20 | **Stroke** | 0.2 | **20** | **Chronic sinusitis** | 0.3 | **20** | **Tuberculosis** | 1.7 | **20** | **Chronic sinusitis** | 1.7 |
| 21 | **Tuberculosis** | 0.2 | **21** | **Parkinson’s disease** | 0.3 | **21** | **Meningitis** | 1.4 | **21** | **Tuberculosis** | 1.4 |
| 22 | **Anaemia** | 0.1 | **22** | **Chronic obstructive pulmonary disease** | 0.2 | **22** | **Thyroid problem** | 0.9 | **22** | **Multiple sclerosis** | 1.2 |
| 23 | **Bronchiectasis** | 0.1 | **23** | **Meningitis** | 0.2 | **23** | **Bronchiectasis** | 0.6 | **23** | **Bronchiectasis** | 1.0 |
| 24 | **Hepatitis** | 0.1 | **24** | **Tuberculosis** | 0.2 | **24** | **Chronic obstructive pulmonary disease** | 0.6 | **24** | **Meningitis** | 1.0 |
| 25 | **Multiple sclerosis** | 0.1 | **25** | **Anaemia** | 0.1 | **25** | **Anaemia** | 0.5 | **25** | **Parkinson’s disease** | 0.8 |
| 26 | **Peripheral vascular disease** | 0.1 | **26** | **Bronchiectasis** | 0.1 | **26** | **Multiple sclerosis** | 0.5 | **26** | **Peripheral vascular disease** | 0.7 |
| 27 | **Prostate problem** | 0.1 | **27** | **Cirrhosis** | 0.1 | **27** | **Peripheral vascular disease** | 0.5 | **27** | **Thyroid problem** | 0.7 |
| 28 | **Thyroid problem** | 0.1 | **28** | **Peripheral vascular disease** | 0.1 | **28** | **Prostate problem** | 0.5 | **28** | **Anaemia** | 0.6 |
| 29 | **Chronic kidney disease** | 0.0 | **29** | **Prostate problem** | 0.1 | **29** | **Hepatitis** | 0.4 | **29** | **Prostate problem** | 0.5 |
| 30 | **Chronic obstructive pulmonary disease** | 0.0 | **30** | **Schizophrenia** | 0.1 | **30** | **Parkinson’s disease** | 0.2 | **30** | **Schizophrenia** | 0.5 |
| 31 | **Cirrhosis** | 0.0 | **31** | **Thyroid problem** | 0.1 | **31** | **Schizophrenia** | 0.2 | **31** | **Cirrhosis** | 0.4 |
| 32 | **Dementia** | 0.0 | **32** | **Chronic kidney disease** | 0.0 | **32** | **Chronic kidney disease** | 0.1 | **32** | **Heart failure** | 0.4 |
| 33 | **Heart failure** | 0.0 | **33** | **Dementia** | 0.0 | **33** | **Cirrhosis** | 0.1 | **33** | **Chronic kidney disease** | 0.3 |
| 34 | **Inflammatory bowel disease** | 0.0 | **34** | **Heart failure** | 0.0 | **34** | **Heart failure** | 0.1 | **34** | **Hepatitis** | 0.2 |
| 35 | **Parkinson’s disease** | 0.0 | **35** | **Hepatitis** | 0.0 | **35** | **Inflammatory bowel disease** | 0.1 | **35** | **Dementia** | 0.1 |
| 36 | **Schizophrenia** | 0.0 | **36** | **Inflammatory bowel disease** | 0.0 | **36** | **Dementia** | 0.0 | **36** | **Inflammatory bowel disease** | 0.1 |

**Table S4.** Participant characteristics by overall health status and multimorbidity

| Characteristics | Status | | | | |
| --- | --- | --- | --- | --- | --- |
|  | Good health & without multimorbidity  (n = 317,264) | Poor health & without multimorbidity  (n = 76,495) | Good health &  with multimorbidity  (n = 48,588) | Poor health &  with multimorbidity  (n = 47,268) | P-value |
| Sex |  |  |  |  |  |
| Female | 177,451 (55.9) | 37,801 (49.4) | 27,630 (56.9) | 23,757 (50.3) |  |
| Male | 139,813 (44.1) | 38,694 (50.6) | 20,958 (43.1) | 23,511 (49.7) | <0.001 |
| Age, years (median [IQR]) | 57 [49-63] | 56 [48-62] | 61 [55-65] | 60 [54-65] |  |
| Age categories |  |  |  |  |  |
| ≤ 50 | 90,217 (28.4) | 24,236 (31.7) | 7,010 (14.4) | 7,629 (16.1) |  |
| 51-60 | 113,659 (35.8) | 27,883 (36.5) | 15,025 (30.9) | 16,358 (34.6) |  |
| > 60 | 113,388 (35.7) | 24,376 (31.9) | 26,553 (54.6) | 23,281 (49.3) | <0.001 |
| Ethnicity |  |  |  |  |  |
| White | 302,015 (95.2) | 69,687 (91.1) | 46,485 (95.7) | 43,887 (92.8) |  |
| Non-white | 14,303 (4.5) | 6,496 (8.5) | 1,942 (4.0) | 3,178 (6.7) |  |
| Missing | 946 (0.3) | 312 (0.4) | 161 (0.3) | 203 (0.4) | <0.001 |
| Socioeconomic status |  |  |  |  |  |
| 1^st^ quintile (least deprived) | 69,942 (22.0) | 11,903 (15.6) | 10,314 (21.2) | 6,771 (14.3) |  |
| 2^nd^ quintile | 67,625 (21.3) | 12,853 (16.8) | 10,213 (21.0) | 7,456 (15.8) |  |
| 3^rd^ quintile | 65,585 (20.7) | 14,208 (18.6) | 9,914 (20.4) | 8,345 (17.7) |  |
| 4^th^ quintile | 61,727 (19.5) | 16,655 (21.8) | 9,485 (19.5) | 10,020 (21.2) |  |
| 5^th^ quintile (most deprived) | 52,026 (16.4) | 20,749 (27.1) | 8,602 (17.7) | 14,613 (30.9) |  |
| Missing | 359 (0.1) | 127 (0.2) | 60 (0.1) | 63 (0.1) | <0.001 |
| Education level |  |  |  |  |  |
| College or University degree | 115,302 (36.3) | 18,927 (24.7) | 15,491 (31.9) | 10,001 (21.2) |  |
| A/AS level or equivalent | 37,486 (11.8) | 7,520 (9.8) | 5,369 (11.1) | 4,365 (9.2) |  |
| O Levels/ GCSEs or equivalent | 84,293 (26.6) | 22,047 (28.8) | 12,078 (24.9) | 11,934 (25.2) |  |
| Other (e.g. NVQ, nursing, missing) | 80,183 (25.3) | 28,001 (36.6) | 15,650 (32.2) | 20,968 (44.4) | <0.001 |
| Employment status |  |  |  |  |  |
| Working | 199,904 (63.0) | 43,355 (56.7) | 22,561 (46.4) | 16,470 (34.8) |  |
| Retired | 97,679 (30.8) | 21,299 (27.8) | 22,782 (46.9) | 20,793 (44.0) |  |
| Unemployed | 4,186 (1.3) | 2,196 (2.9) | 594 (1.2) | 1,013 (2.1) |  |
| Other (student, volunteer/ missing) | 15,495 (4.9) | 9,645 (12.6) | 2,651 (5.5) | 8,992 (19.0) | <0.001 |
| BMI categories, kg/m^2^ |  |  |  |  |  |
| Underweight (<18.5) | 1,594 (0.5) | 472 (0.6) | 184 (0.4) | 239 (0.5) |  |
| Normal weight (18.5-24.9) | 119,831 (37.8) | 17,757 (23.2) | 13,609 (28.0) | 7,835 (16.6) |  |
| Overweight (25.0-29.9) | 139,047 (43.8) | 30,177 (39.4) | 21,455 (44.2) | 16,834 (35.6) |  |
| Obese (≥30.0) | 55,742 (17.6) | 27,439 (35.9) | 13,161 (27.1) | 21,948 (46.4) |  |
| Missing | 1,050 (0.3) | 650 (0.8) | 179 (0.4) | 412 (0.9) | <0.001 |
| Smoking status |  |  |  |  |  |
| Never | 184,725 (58.2) | 37,037 (48.4) | 25,600 (52.7) | 20,622 (43.6) |  |
| Former | 104,640 (33.0) | 25,565 (33.4) | 19,347 (39.8) | 19,435 (41.1) |  |
| Current | 26,970 (8.5) | 13,530 (17.7) | 3,450 (7.1) | 6,940 (14.7) |  |
| Missing | 929 (0.3) | 363 (0.5) | 191 (0.4) | 271 (0.6) | <0.001 |
| Alcohol consumption |  |  |  |  |  |
| Never or <14 units/week | 192,644 (60.7) | 49,922 (65.3) | 30,983 (63.8) | 32,846 (69.5) |  |
| Excess ≥14 units/week | 124,358 (39.2) | 26,409 (34.5) | 17,553 (36.1) | 14,340 (30.3) |  |
| Missing | 262 (0.1) | 164 (0.2) | 52 (0.1) | 82 (0.2) | <0.001 |
| Meet fruit/vegetable guidelines |  |  |  |  |  |
| No | 192,108 (60.6) | 52,947 (69.2) | 27,691 (57.0) | 30,489 (64.5) |  |
| Yes | 124,921 (39.4) | 23,380 (30.6) | 20,863 (42.9) | 16,706 (35.3) |  |
| Missing | 235 (0.1) | 168 (0.2) | 34 (0.1) | 73 (0.2) | <0.001 |
| Oily fish (≥1/week) |  |  |  |  |  |
| No | 137,630 (43.4) | 38,783 (50.7) | 18,784 (38.7) | 21,805 (46.1) |  |
| Yes | 179,536 (56.6) | 37,667 (49.2) | 29,790 (61.3) | 25,448 (53.8) |  |
| Missing | 98 (0.0) | 45 (0.1) | 14 (0.0) | 15 (0.0) | <0.001 |
| Non-oily fish (≥1/week) |  |  |  |  |  |
| No | 105,864 (33.4) | 28,933 (37.8) | 15,114 (31.1) | 16,503 (34.9) |  |
| Yes | 211,318 (66.6) | 47,520 (62.1) | 33,465 (68.9) | 30,751 (65.1) |  |
| Missing | 82 (0.0) | 42 (0.1) | 9 (0.0) | 14 (0.0) | <0.001 |
| Processed meat (≥2/week) |  |  |  |  |  |
| No | 225,616 (71.1) | 47,901 (62.6) | 34,690 (71.4) | 30,003 (63.5) |  |
| Yes | 91,584 (28.9) | 28,553 (37.3) | 13,886 (28.6) | 17,249 (36.5) |  |
| Missing | 64 (0.0) | 41 (0.1) | 12 (0.0) | 16 (0.0) | <0.001 |
| Red meat (≥2/week) |  |  |  |  |  |
| No | 272,289 (85.8) | 63,752 (83.3) | 41,267 (84.9) | 39,118 (82.8) |  |
| Yes | 44,938 (14.2) | 12,724 (16.6) | 7,315 (15.1) | 8,144 (17.2) |  |
| Missing | 37 (0.0) | 19 (0.0) | 6 (0.0) | 6 (0.0) | <0.001 |
| Sedentary behaviour, hours |  |  |  |  |  |
| Low (<4) | 153,111 (48.3) | 29,884 (39.1) | 21,265 (43.8) | 16,668 (35.3) |  |
| Moderate (4-6) | 106,706 (33.6) | 25,993 (34.0) | 17,048 (35.1) | 16,085 (34.0) |  |
| High (≥6) | 57,255 (18.0) | 20,499 (26.8) | 10,225 (21.0) | 14,432 (30.5) |  |
| Missing | 192 (0.1) | 119 (0.2) | 50 (0.1) | 83 (0.2) | <0.001 |
| LTPA, MET-mins/week, (median [IQR]) | 731 [287-1523] | 363 [86-911] | 674 [242-1428] | 286 [49-838] | <0.001 |
| LTPA categories, MET-mins/week |  |  |  |  |  |
| Low (<600) | 136,315 (43.0) | 47,990 (62.7) | 22,253 (45.8) | 30,903 (65.4) |  |
| Moderate (600 to <3000) | 153,690 (48.4) | 24,666 (32.3) | 22,503 (46.3) | 14,047 (29.7) |  |
| High (≥3000) | 25,825 (8.1) | 3,000 (3.9) | 3,532 (7.3) | 1,639 (3.5) |  |
| Missing | 1,434 (0.5) | 839 (1.1) | 300 (0.6) | 679 (1.4) | <0.001 |
| TPA MET-mins/week, (median [IQR]) | 1812 [855-3546] | 1299 [532-2880] | 1733 [792-3466] | 1188 [490-2733] | <0.001 |
| TPA categories, MET-mins/week |  |  |  |  |  |
| Low (<600) | 50,740 (16.0) | 18,770 (24.5) | 8,406 (17.3) | 12,240 (25.9) |  |
| Moderate (600 to <3000) | 156,567 (49.4) | 32,607 (42.6) | 23,449 (48.3) | 19,408 (41.1) |  |
| High (≥3000) | 92,025 (29.0) | 16,144 (21.1) | 13,555 (27.9) | 8,985 (19.0) |  |
| Missing | 17,932 (5.7) | 8,974 (11.7) | 3,178 (6.5) | 6,635 (14.0) | <0.001 |
| Objective m*g*, (median [IQR]) ^a^ | 27.9 [23.4-33.4] | 25.3 [20.8-30.5] | 25.5 [21.2-30.8] | 22.9 [18.4-27.8] | <0.001 |
| Objective categories, mins/day of brisk walking ^a^ | |  |  |  |  |
| Low (4) | 19,697 (28.7) | 4,793 (42.8) | 3,826 (40.9) | 3,503 (56.7) |  |
| Moderate (10) | 23,487 (30.2) | 3,591 (32.1) | 3,052 (32.6) | 1,687 (27.3) |  |
| High (22) | 25,516 (37.1) | 2,806 (25.1) | 2,479 (26.5) | 992 (16.1) | <0.001 |

Shown are numbers (%) unless stated otherwise.

**^a^** Data taken at the time of objective physical activity measurements.

P-value indicates the difference between categories (Chi-square test); continuous data (Wilcoxon rank-sum test).

A/AS level or equivalent= Higher School Certificate; O Levels/GCSEs=School Certificate; NVQ= National Vocational Qualification; BMI=body mass index; LTPA=leisure-time physical activity questionnaire; TPA=total physical activity questionnaire; MET=metabolic equivalent of task, mg=milli-gravitational units.

# Table S5. Number of participants with the top-10 comorbidity

|  | CHD (MI) | Hypertension | Heart failure | Stroke | Atrial fibrillation | Diabetes | COPD | Arthritis | Depression | Dementia |
| --- | --- | --- | --- | --- | --- | --- | --- | --- | --- | --- |
| CHD (MI) | - |  |  |  |  |  |  |  |  |  |
| Hypertension | 5,861 | - |  |  |  |  |  |  |  |  |
| Heart failure | 55 | 156 | - |  |  |  |  |  |  |  |
| Stroke | 730 | 3,563 | 22 | - |  |  |  |  |  |  |
| Atrial fibrillation | 221 | 1,545 | 46 | 186 | - |  |  |  |  |  |
| Diabetes | 1,758 | 13,424 | 52 | 838 | 252 | - |  |  |  |  |
| COPD | 118 | 579 | 9 | 46 | 27 | 103 | - |  |  |  |
| Arthritis | 207 | 1,878 | 5 | 134 | 46 | 337 | 37 | - |  |  |
| Depression | 658 | 8,030 | 30 | 570 | 154 | 1,370 | 187 | 367 | - |  |
| Dementia | 6 | 31 | 0 | 3 | 1 | 2 | 0 | 3 | 14 | - |

CHD=coronary heart disease; MI= myocardial infarction; COPD=chronic obstructive pulmonary disease.

**Table S6.** Characteristics by participants with and without top-10 comorbidity

| Characteristics | Status | | |
| --- | --- | --- | --- |
|  | With top-10 comorbidity  (n = 32,876) | Without top-10 comorbidity  (n = 459,063) | P-value |
| Sex |  |  |  |
| Female | 13,557 (41.2) | 254,326 (55.4) |  |
| Male | 19,319 (58.8) | 204,737 (44.6) | <0.001 |
| Age, years (median [IQR]) | 62 [56-66] | 57 [50-63] | <0.001 |
| Age categories |  |  |  |
| ≤ 50 | 3553 (10.8) | 126,279 (27.5) |  |
| 51-60 | 10,682 (32.5) | 163,030 (35.5) |  |
| > 60 | 18,641 (56.7) | 169,754 (37.0) | <0.001 |
| Ethnicity |  |  |  |
| White | 30,218 (91.9) | 433,586 (94.5) |  |
| Non-white | 2505 (7.6) | 23,896 (5.2) |  |
| Missing | 153 (0.5) | 1,581 (0.3) | <0.001 |
| Socioeconomic status |  |  |  |
| 1^st^ quintile (least deprived) | 4,841 (14.7) | 94,337 (20.5) |  |
| 2^nd^ quintile | 5,542 (16.9) | 92,876 (20.2) |  |
| 3^rd^ quintile | 5,933 (18.0) | 92,468 (20.1) |  |
| 4^th^ quintile | 6,794 (20.7) | 91,531 (19.9) |  |
| 5^th^ quintile (most deprived) | 9,721 (29.6) | 87,280 (19.0) |  |
| Missing | 45 (0.1) | 571 (0.1) | <0.001 |
| Education level |  |  |  |
| College or University degree | 7,222 (22.0) | 152,996 (33.3) |  |
| A/AS level or equivalent | 3,019 (9.2) | 51,905 (11.3) |  |
| O Levels/ GCSEs or equivalent | 8,023 (24.4) | 122,790 (26.7) |  |
| Other (e.g. NVQ, nursing, missing) | 14,612 (44.4) | 131,372 (28.6)) | <0.001 |
| Employment status |  |  |  |
| Working | 10,981 (33.4) | 272,170 (59.3) |  |
| Retired | 16,042 (48.8) | 147,196 (32.1) |  |
| Unemployed | 665 (2.0) | 7,447 (1.6) |  |
| Other (student, volunteer/ missing) | 5,188 (15.8) | 32,250 (7.0) | <0.001 |
| BMI categories, kg/m^2^ |  |  |  |
| Underweight (<18.5) | 4,294 (13.1) | 155,302 (33.8) |  |
| Normal weight (18.5-24.9) | 12,014 (36.5) | 196,361 (42.8) |  |
| Overweight (25.0-29.9) | 16,227 (49.4) | 102,854 (22.4) |  |
| Obese (≥30.0) | 65 (0.2) | 2,450 (0.5) |  |
| Missing | 276 (0.8) | 2,096 (0.5) | <0.001 |
| Smoking status |  |  |  |
| Never | 13,826 (42.1) | 255,224 (55.6) |  |
| Former | 14,697 (44.7) | 154,996 (33.8) |  |
| Current | 4,142 (12.6) | 47,200 (10.3) |  |
| Missing | 211 (0.6) | 1,643 (0.4) | <0.001 |
| Alcohol consumption |  |  |  |
| Never or <14 units/week | 21,939 (66.7) | 286,090 (62.3) |  |
| Excess ≥14 units/week | 10,865 (33.0) | 172,384 (37.6) |  |
| Missing | 72 (0.2) | 589 (0.1) | <0.001 |
| Meet fruit/vegetable guidelines |  |  |  |
| No | 20,131 (61.2) | 284,578 (62.0) |  |
| Yes | 12,693 (38.6) | 173,935 (37.9) |  |
| Missing | 52 (0.2) | 550 (0.1) | <0.001 |
| Oily fish (≥1/week) |  |  |  |
| No | 14,017 (42.6) | 204,075 (44.5) |  |
| Yes | 18,847 (57.3) | 254,770 (55.5) |  |
| Missing | 12 (0.0) | 218 (0.0) | <0.001 |
| Non-oily fish (≥1/week) |  |  |  |
| No | 10,928 (33.2) | 156,372 (34.1) |  |
| Yes | 21,937 (66.7) | 302,498 (65.9) |  |
| Missing | 11 (0.0) | 193 (0.0) | <0.001 |
| Processed meat (≥2/week) |  |  |  |
| No | 20,935 (63.7) | 318,788 (69.4) |  |
| Yes | 11,925 (36.3) | 140,097 (30.9) |  |
| Missing | 16 (0.0) | 178 (0.0) | <0.001 |
| Red meat (≥2/week) |  |  |  |
| No | 26,990 (82.1) | 391,292 (85.2) |  |
| Yes | 5,881 (17.9) | 676,61 (14.7) |  |
| Missing | 5 (0.0) | 110 (0.0) | <0.001 |
| Sedentary behaviour, hours |  |  |  |
| Low (<4) | 11,143 (33.9) | 210,938 (45.9) |  |
| Moderate (4-6) | 11,356 (34.5) | 155,079 (33.8) |  |
| High (≥6) | 10,307 (31.4) | 92,604 (20.2) |  |
| Missing | 70 (0.2) | 442 (0.1) | <0.001 |
| LTPA, MET-mins/week, (median [IQR]) | 394 [75-1069] | 636 [218-1376] | <0.001 |
| LTPA categories, MET-mins/week |  |  |  |
| Low (<600) | 219,393 (47.8) | 19,578 (59.6) |  |
| Moderate (600 to <3000) | 204,280 (44.5) | 11,237 (34.2) |  |
| High (≥3000) | 32,477 (7.1) | 1,609 (4.9) |  |
| Missing | 2,913 (0.6) | 452 (1.4) | <0.001 |
| TPA MET-mins/week, (median [IQR]) | 1359 [556-2910] | 1680 [756-3390] | <0.001 |
| TPA categories, MET-mins/week |  |  |  |
| Low (<600) | 82,879 (18.1) | 7,819 (23.8) |  |
| Moderate (600 to <3000) | 218,742 (47.7) | 14,114 (42.9) |  |
| High (≥3000) | 124,096 (27.0) | 6,989 (21.3) |  |
| Missing | 33,346 (7.3) | 3,954 (12.0) | <0.001 |
| Objective m*g*, (median [IQR]) ^a^ | 22.3 [18.1-27.2] | 27.3 [22.7-32.7] | <0.001 |
| Objective categories, mins/day of brisk walking ^a^ | |  |  |
| Low (4) | 29,280 (32.1) | 2,639 (60.0) |  |
| Moderate (10) | 30,734 (33.7) | 1,137 (25.9) |  |
| High (22) | 31,206 (34.2) | 620 (14.1) | <0.001 |

Shown are numbers (%) unless stated otherwise.

**^a^** Data taken at the time of objective physical activity measurements.

P-value indicates the difference between categories (Chi-square test); continuous data (Wilcoxon rank-sum test).

A/AS level or equivalent= Higher School Certificate; O Levels/GCSEs=School Certificate; NVQ= National Vocational Qualification; BMI=body mass index; LTPA=leisure-time physical activity questionnaire; TPA=total physical activity questionnaire; MET=metabolic equivalent of task, mg=milli-gravitational units.

# Table S7. Participant characteristics who undertook the objective physical activity by multimorbidity status

| Characteristics | Multimorbidity status | | |
| --- | --- | --- | --- |
|  | With multimorbidity  (n = 15,607) | Without multimorbidity  (n = 80,009) | P-value |
| Sex |  |  |  |
| Female | 8,566 (54.9) | 45,260 (56.6) |  |
| Male | 7,041 (45.1) | 34,749 (43.4) | <0.001 |
| Age, years (median [IQR]) ^a^ | 65.7 [59.5-69.9] | 62.3 [55.2-67.6] | <0.001 |
| Age categories ^a^ |  |  |  |
| ≤ 50 | 833 (5.3) | 8,420 (10.5) |  |
| 51-60 | 3,323 (21.3) | 24,510 (30.6) |  |
| > 60 | 11,451 (73.4) | 47,079 (58.8) | <0.001 |
| Ethnicity |  |  |  |
| White | 15,071 (96.6) | 77,296 (96.6) |  |
| Non-white | 474 (3.0) | 2,487 (3.1) |  |
| Missing | 62 (0.4) | 226 (0.3) | 0.016 |
| Socioeconomic status |  |  |  |
| 1^st^ quintile (least deprived) | 3,318 (21.3) | 18,476 (23.1) |  |
| 2^nd^ quintile | 3,277 (21.0) | 17,453 (21.8) |  |
| 3^rd^ quintile | 3,185 (20.4) | 16,505 (20.6) |  |
| 4^th^ quintile | 3,114 (20.0) | 15,593 (19.5) |  |
| 5^th^ quintile (most deprived) | 2,697 (17.3) | 11,890 (14.9) |  |
| Missing | 16 (0.1) | 92 (0.1) | <0.001 |
| Education level |  |  |  |
| College or University degree | 5,886 (37.7) | 35,503 (44.4) |  |
| A/AS level or equivalent | 1,982 (12.7) | 10,618 (13.3) |  |
| O Levels/ GCSEs or equivalent | 3,892 (24.9) | 19,472 (24.3) |  |
| Other (e.g. NVQ, nursing, missing) | 3,847 (24.6) | 14,416 (18.0) | <0.001 |
| Employment status |  |  |  |
| Working | 7,465 (47.8) | 51,556 (64.4) |  |
| Retired | 6,576 (42.1) | 23,313 (29.1) |  |
| Unemployed | 203 (1.3) | 908 (1.1) |  |
| Other (student, volunteer/ missing) | 1,363 (8.7) | 4,232 (5.3) | <0.001 |
| BMI categories, kg/m^2^ |  |  |  |
| Underweight (<18.5) | 4,295 (27.5) | 32,738 (40.9) |  |
| Normal weight (18.5-24.9) | 6,314 (40.5) | 33,027 (41.3) |  |
| Overweight (25.0-29.9) | 4,876 (31.2) | 13,646 (17.1) |  |
| Obese (≥30.0) | 78 (0.5) | 458 (0.6) |  |
| Missing | 44 (0.3) | 140 (0.2) | <0.001 |
| Smoking status |  |  |  |
| Never | 7,962 (51.0) | 46,505 (58.1) |  |
| Former | 6,530 (41.8) | 27,809 (34.8) |  |
| Current | 1,071 (6.9) | 5,530 (6.9) |  |
| Missing | 44 (0.3) | 165 (0.2) | <0.001 |
| Alcohol consumption |  |  |  |
| Never or <14 units/week | 9,914 (63.5) | 48,178 (60.2) |  |
| Excess ≥14 units/week | 5,682 (36.4) | 31,791 (39.7) |  |
| Missing | 11 (0.1) | 40 (0.0) | <0.001 |
| Meet fruit/vegetable guidelines |  |  |  |
| No | 9,170 (58.8) | 47,921 (59.9) |  |
| Yes | 6,430 (41.2) | 32,063 (40.1) |  |
| Missing | 7 (0.0) | 25 (0.0) | <0.001 |
| Oily fish (≥1/week) |  |  |  |
| No | 6,408 (41.1) | 35,209 |  |
| Yes | 9,198 (58.9) | 44,794 |  |
| Missing | 1 (0.0) | 6 | <0.001 |
| Non-oily fish (≥1/week) |  |  |  |
| No | 5,318 (34.1) | 28,198 (35.2) |  |
| Yes | 10,289 (65.9) | 51,805 (64.7) |  |
| Missing | 0 (0.0) | 6 (0.0) | <0.001 |
| Processed meat (≥2/week) |  |  |  |
| No | 10,836 (69.4) | 57,117 (71.4) |  |
| Yes | 4,770 (30.6) | 22,882 (28.6) |  |
| Missing | 1 (0.0) | 10 (0.0) | <0.001 |
| Red meat (≥2/week) |  |  |  |
| No | 13,316 (85.3) | 69,472 (86.8) |  |
| Yes | 2,291 (14.7) | 10,533 (13.2) |  |
| Missing | 0 (0.0) | 4 (0.0) | <0.001 |
| Sedentary behaviour, hours |  |  |  |
| Low (<4) | 6,205 (39.8) | 38,714 (48.4) |  |
| Moderate (4-6) | 5,484 (35.1) | 26,841 (33.5) |  |
| High (≥6) | 3,908 (25.0) | 14,432 (18.0) |  |
| Missing | 10 (0.1) | 22 (0.0) | <0.001 |
| LTPA, MET-mins/week, (median [IQR]) | 619 [197-1336] | 755 [323-1534] | <0.001 |
| LTPA categories, MET-mins/week |  |  |  |
| Low (<600) | 33,093 (41.4) | 7,593 (48.7) |  |
| Moderate (600 to <3000) | 40,146 (50.5) | 6,925 (44.4) |  |
| High (≥3000) | 6,275 (7.8) | 1,021 (6.5) |  |
| Missing | 225 (0.3) | 68 (0.4) | <0.001 |
| TPA MET-mins/week, (median [IQR]) | 1506 [693-3035] | 1708 [818-3252] | <0.001 |
| TPA categories, MET-mins/week |  |  |  |
| Low (<600) | 13,554 (16.9) | 3,143 (20.1) |  |
| Moderate (600 to <3000) | 41,502 (51.9) | 7,736 (49.6) |  |
| High (≥3000) | 21,278 (26.6) | 3,665 (23.5) |  |
| Missing | 3,675 (4.6) | 1,063 (6.8) | <0.001 |
| Objective m*g*, (median [IQR]) ^a^ | 24.5 [20.1-29.7] | 27.6 [22.9-33.0] | <0.001 |
| Objective categories, mins/day of brisk walking ^a^ | |  |  |
| Low (4) | 24,550 (30.7) | 7,369 (47.2) |  |
| Moderate (10) | 27,114 (33.9) | 4,757 (30.5) |  |
| High (22) | 28,345 (35.4) | 3,481 (22.3) | <0.001 |

Shown are numbers (%) unless stated otherwise.

**^a^** Data taken at the time of objective physical activity measurements.

P-value indicates the difference between categories (Chi-square test); continuous data (Wilcoxon rank-sum test).

A/AS level or equivalent= Higher School Certificate; O Levels/GCSEs=School Certificate; NVQ= National Vocational Qualification; BMI=body mass index; LTPA=leisure-time physical activity questionnaire; TPA=total physical activity questionnaire; MET=metabolic equivalent of task, mg=milli-gravitational units.

# Table S8. Participants characteristics by completion of the objective physical activity measurements

| Characteristics | Completed the objective PA measurements | | |
| --- | --- | --- | --- |
|  | Yes  (n = 95,616) | No  (n = 396,323) | P-value |
| Sex |  |  |  |
| Female | 53,826 (56.3) | 214,057 (54.0) |  |
| Male | 41,790 (43.7) | 182,266 (46.0) | <0.001 |
| Age, years (median [IQR]) | 63 [56-68] ^a^ | 58 [50-63] | <0.001 |
| Age categories |  |  |  |
| ≤50 | 9,253 (9.7) ^a^ | 104,635 (26.4) |  |
| 51-60 | 27,833 (29.1) ^a^ | 136,912 (34.5) |  |
| > 60 | 58,530 (61.2) ^a^ | 154,776 (39.1) | <0.001 |
| Ethnicity |  |  |  |
| White | 92,367 (96.6) | 371,437 (93.7) |  |
| Non-white | 2,961 (3.1) | 23,440 (5.9) |  |
| Missing | 288 (0.3) | 1,446 (0.4) | <0.001 |
| Socioeconomic status |  |  |  |
| 1^st^ quintile (least deprived) | 21,794 (22.8) | 77,384 (19.5) |  |
| 2^nd^ quintile | 20,730 (21.7) | 77,688 (19.6) |  |
| 3^rd^ quintile | 19,690 (20.6) | 78,711 (19.6) |  |
| 4^th^ quintile | 18,707 (19.6) | 79,618 (20.1) |  |
| 5^th^ quintile (most deprived) | 14,587 (15.3) | 82,414 (20.8) |  |
| Missing | 108 (0.1) | 508 (0.1) | <0.001 |
| Education level |  |  |  |
| College or University degree | 41,389 (43.3) | 118,829 (30.0) |  |
| A/AS level or equivalent | 12,600 (13.2) | 42,324 (10.7) |  |
| O Levels/ GCSEs or equivalent | 23,364 (24.4) | 107,449 (27.1) |  |
| Other (e.g. NVQ, nursing, missing) | 18,263 (19.1) | 127,721 (32.2) | <0.001 |
| Employment status |  |  |  |
| Working | 59,021 (61.7) | 224,130 (56.6) |  |
| Retired | 29,889 (31.2) | 133,349 (33.6) |  |
| Unemployed | 1,111 (1.2) | 7,001 (1.8) |  |
| Other (student, volunteer/ missing) | 5,595 (5.9) | 31,834 (8.0) | <0.001 |
| BMI categories, kg/m^2^ |  |  |  |
| Underweight (<18.5) | 536 (0.6) | 1,979 (0.5) |  |
| Normal weight (18.5-24.9) | 37,033 (38.7) | 122,563 (30.9) |  |
| Overweight (25.0-29.9) | 39,341 (41.1) | 169,034 (42.7) |  |
| Obese (≥30.0) | 18,522 (19.4) | 100,559 (25.4) |  |
| Missing | 184 (0.2) | 2,188 (0.6) | <0.001 |
| Smoking status |  |  |  |
| Never | 54,467 (57.0) | 214,583 (54.1) |  |
| Former | 34,339 (35.9) | 135,354 (34.2) |  |
| Current | 6,601 (6.9) | 44,741 (11.3) |  |
| Missing | 209 (0.2) | 1,645 (0.4) | <0.001 |
| Alcohol consumption |  |  |  |
| Never or <14 units/week | 58,092(60.8) | 249,937 (63.1) |  |
| Excess ≥14 units/week | 37,473 (39.2) | 145,776 (36.8) |  |
| Missing | 51 (0.1) | 610 (0.2) | <0.001 |
| Meet fruit/vegetable guidelines |  |  |  |
| No | 57,091 (59.7) | 247,618 (62.5) |  |
| Yes | 38,493 (40.3) | 148,135 (37.4) |  |
| Missing | 32 (0.0) | 570 (0.1) | <0.001 |
| Oily fish (≥1/week) |  |  |  |
| No | 41,617 (43.5) | 176,475 (44.5) |  |
| Yes | 53,992 (56.5) | 219,625 (55.4) |  |
| Missing | 7 (0.0) | 223 (0.1) | <0.001 |
| Non-oily fish (≥1/week) |  |  |  |
| No | 33,516 (35.1) | 133,784 (33.8) |  |
| Yes | 62,094 (64.9) | 262,341 (66.2) |  |
| Missing | 6 (0.0) | 198 (0.0) | <0.001 |
| Processed meat (≥2/week) |  |  |  |
| No | 67,953 (71.1) | 271,770 (68.6) |  |
| Yes | 27,652 (28.9) | 124,370 (31.4) |  |
| Missing | 11 (0.0) | 183 (0.0) | <0.001 |
| Red meat (≥2/week) |  |  |  |
| No | 82,788 (86.6) | 335,494 (84.7) |  |
| Yes | 12,824 (13.4) | 60,718 (15.3) |  |
| Missing | 4 (0.0) | 111 (0.0) | <0.001 |
| Sedentary behaviour, hours |  |  |  |
| Low (<4) | 44,919 (47.0) | 177,162 (44.7) |  |
| Moderate (4-6) | 32,325 (33.8) | 134,110 (33.8) |  |
| High (≥6) | 18,340 (19.2) | 84,571 (21.3) |  |
| Missing | 32 (0.0) | 480 (0.1) | <0.001 |

Shown are numbers (%) unless stated otherwise. PA=physical activity.

P-value indicates the difference between categories (Chi-square test); continuous data (Wilcoxon rank-sum test).

^a^ Age was calculated at the time of objective PA measurement; all other covariates were obtained at baseline assessment.

A/AS level or equivalent= Higher School Certificate; O Levels/GCSEs=School Certificate; NVQ= National Vocational

Qualification; BMI=body mass index.

# Table S9. Self-reported physical activity in tertiles and years of life gained at the age of 45 years by multimorbidity status

| Characteristics | All participants  (N = 491,939) | Multimorbidity status | | |
| --- | --- | --- | --- | --- |
|  |  | **With multimorbidity**  (n = 96,622) | **Without multimorbidity**  (n = 395,317) | **P-value** |
| Hazard ratio (95%CI) |  |  |  |  |
| LTPA tertiles, MET-mins/week |  |  |  |  |
| Lowest (<332) | 162,940 (33.1) | 39,334 (40.7) | 123,606 (31.3) |  |
| Middle (332-1050) | 162,872 (33.1) | 29,459 (30.5) | 133,413 (33.8) |  |
| Upper (≥1050) | 162,762 (33.1) | 26,824 (27.8) | 135,938 (34.4) |  |
| Missing | 3,365 (0.7) | 1,005 (1.0) | 2,360 (0.6) | <0.001 |
|  |  |  |  |  |
| TPA tertiles, MET-mins/week |  |  |  |  |
| Lowest (<1013) | 151,577 (30.8) | 33,040 (34.2) | 118,537 (30.0) |  |
| Middle (1013-2628) | 151,655 (30.8) | 27,300 (28.3) | 124,355 (31.5) |  |
| Upper (≥2628) | 151,407 (30.8) | 26,258 (27.2) | 125,149 (31.7) |  |
| Missing | 37,300 (7.6) | 10,024 (10.4) | 27,276 (6.9) | <0.001 |
|  |  |  |  |  |
| Years of life gained at 45 years (95% CI) | |  |  |  |
| LTPA tertiles, MET-mins/week |  |  |  |  |
| Moderate vs. low |  | 3.22 (2.56, 3.88) | 1.93 (1.51, 2.36) |  |
| High vs. low |  | 4.10 (3.40, 4.79) | 2.45 (2.02, 2.88) |  |
|  |  |  |  |  |
| TPA tertiles, MET-mins/week |  |  |  |  |
| Moderate vs. low |  | 1.65 (0.98, 2.33) | 1.39 (0.96, 1.83) |  |
| High vs. low |  | 2.28 (1.58, 2.97) | 1.23 (0.81, 1.66) |  |

Shown are numbers (%) unless stated otherwise. CI=confidence intervals

P-value indicates the difference between categories (Chi-square test).

LTPA=leisure-time physical activity questionnaire; TPA=total physical activity questionnaire; MET=metabolic equivalent of task.

# Table S10. Association between continuously measured (log-transformed) physical activity and mortality

| Physical activity | Hazard Ratio (95% CI) ^a^ | |
| --- | --- | --- |
|  | **With multimorbidity** | **Without multimorbidity** |
| Leisure-time TPA | 0.99 (0.99, 0.99) | 0.99 (0.99, 0.99) |
| Total PA | 0.99 (0.99, 1.00) | 0.99 (0.99, 1.00) |
| Objective PA | 0.84 (0.79, 0.89) | 0.77 (0.74, 0.81) |

CI=confidence interval; PA=physical activity

^a^ Hazard ratio represents the risk of all-cause mortality using a log base of 1.1 (i.e. log1.1 (total PA)) to represent a 10% increase in total PA

Fully adjusted model including age, sex, ethnicity, socioeconomic status, employment status, education level, body mass index, smoking status, alcohol consumption, fruit and vegetable, oily fish, non-oily fish, processed meat, red meat intake, and sedentary time.

Self-reported physical activity

Low physical activity group (<600 MET-mins/week) was the reference group (1)

Moderate physical activity group (600 to <3000 MET-mins/week)

High physical activity group (≥3000 MET-mins/week)

Leisure-time physical activity: with multimorbidity (n=95,617; deaths=4,326), and without multimorbidity (n=392,959; deaths=7,011)

Total physical activity: with multimorbidity (n=86,598; deaths=3,758), and without multimorbidity (n= 368,043; deaths=6,458)

Objective physical activity.

Lowest PA (4 mins/day of brisk walking for exercise was the reference group (1)

Moderate PA (10 mins/day of brisk walking for exercise)

High PA (22 mins/day of brisk walking for exercise)

With multimorbidity (n=15,607; deaths=106) and without multimorbidity (n=80,009; deaths=200)

# Table S11. Association between physical activity and mortality for participants with good or poor overall health rating and multimorbidity status, and years of life gained at the age of 45 years

| Physical activity | Hazard ratio (95% CI) | | | | | | | |
| --- | --- | --- | --- | --- | --- | --- | --- | --- |
|  | **Good health &**  **without multimorbidity** | | **Poor health &**  **without multimorbidity** | | **Good health &**  **with multimorbidity** | | **Poor health &**  **with multimorbidity** | |
|  | **Moderate** | **High** | **Moderate** | **High** | **Moderate** | **High** | **Moderate** | **High** |
| Hazard ratio (95%CI) | |  |  |  |  |  |  |  |
| Leisure-time PA | 0.87 (0.82, 0.92) | 0.85 (0.76, 0.94) | 0.81 (0.73, 0.89) | 0.73 (0.58, 0.91) | 0.80 (0.72, 0.90) | 0.77 (0.63, 0.95) | 0.80 (0.74, 0.88) | 0.69 (0.55, 0.86) |
| Total PA | 0.89 (0.82, 0.96) | 0.89 (0.82, 0.97) | 0.84 (0.75, 0.93) | 0.80 (0.70, 0.90) | 0.88 (0.76, 1.01) | 0.90 (0.77, 1.05) | 0.89 (0.81, 0.98) | 0.79 (0.70, 0.88) |
| Objective PA | 0.36 (0.24, 0.54) | 0.29 (0.18, 0.46) | 0.65 (0.32, 1.32) | 0.37 (0.12, 1.09) | 0.46 (0.22, 0.96) | 0.30 (0.11, 0.82) | 0.57 (0.28, 1.14) | 0.31 (0.09, 1.03) |
|  |  |  |  |  |  |  |  |  |
| Years of life gained at 45 years (95% CI) | |  |  |  |  |  |  |  |
| Leisure-time PA | 1.08 (0.68, 1.49) | 0.77 (0.06, 1.48) | 1.92 (1.14, 2.70) | 1.67 (-0.12, 3.46) | 1.57 (0.74, 2.40) | 1.10 (-0.43, 2.63) | 2.21 (1.40, 3.03) | 2.82 (0.83, 4.81) |
| Total PA | 1.11 (0.55, 1.66) | 0.90 (0.31, 1.50) | 1.64 (0.74, 2.53) | 1.60 (0.55, 2.64) | 1.12 (0.02, 2.21) | 0.77 (-0.41, 1.96) | 1.44 (0.56, 2.31) | 2.61 (1.50, 3.72) |
| Objective PA | 4.39 (1.70, 7.08) | 4.77 (1.90, 7.64) | 1.28 (-1.41, 3.96) | 2.18 (-1.70, 6.07) | 3.25 (-1.47, 7.96) | 4.07 (-1.59, 9.73) | 2.31 (-1.72, 6.34) | 4.10 (-2.93, 11.13) |

CI=confidence interval; PA=physical activity

Fully adjusted model including age, sex, ethnicity, socioeconomic status, employment status, education level, body mass index, smoking status, alcohol consumption, fruit and vegetable, oily fish, non-oily fish, processed meat, red meat intake, and sedentary time.

Leisure-time physical activity

Low physical activity group (<600 MET-mins/week) was the reference group (1); Moderate physical activity group (600 to <3000 MET-mins/week); High physical activity group (≥3000 MET-mins/week)

Good health & without multimorbidity (n=315,830; deaths=4,815); Poor health & without multimorbidity (n=75,656; deaths=2,153)

Good health & with multimorbidity (n=48,288; deaths=1,488); Poor health & with multimorbidity (n=46,589; deaths=2,780)

Total physical activity

Low physical activity group (<600 MET-mins/week) was the reference group (1); Moderate physical activity group (600 to <3000 MET-mins/week); High physical activity group (≥3000 MET-mins/week)

Good health & without multimorbidity (n=299,332; deaths=4,552); Poor health & without multimorbidity (n=67,521; deaths=1,868)

Good health & with multimorbidity (n=45,410; deaths=1,401); Poor health & with multimorbidity (n=40,633; deaths=2,316)

Objective physical activity

Low physical activity group (4 mins/day of brisk walking) was the reference group (1); Moderate physical activity group (10 mins/day brisk walking); High physical activity group (22 mins/day of brisk walking)

Good health & without multimorbidity (n=68,700; deaths=154); Poor health & without multimorbidity (n=11,190; deaths=46)

Good health & with multimorbidity (n=9,357; deaths=45); Poor health & with multimorbidity (n=6,182; deaths=61)

# Table S12. Association between physical activity and mortality for participants with and without top-10 comorbidity, and years of life gained at the age of 45 years

| Physical activity | Hazard Ratio (95% CI) | | | |
| --- | --- | --- | --- | --- |
|  | **With top-10 comorbidity** | | **Without top-10 comorbidity** | |
|  | Moderate | High | Moderate | High |
| Hazard ratio (95%CI) |  |  |  |  |
| Leisure-time PA | 0.80 (0.72, 0.88) | 0.71 (0.57, 0.88) | 0.77 (0.74, 0.81) | 0.70 (0.64, 0.76) |
| Total PA | 0.86 (0.77, 0.96) | 0.81 (0.71, 0.93) | 0.83 (0.79, 0.88) | 0.79 (0.74, 0.84) |
| Objective PA | 0.76 (0.38, 1.52) | 0.40 (0.12, 1.38) | 0.38 (0.28, 0.52) | 0.28 (0.19, 0.41) |
|  |  |  |  |  |
| Years of life gained at 45 years (95% CI) | |  |  |  |
| Leisure-time PA | 2.32 (2.00, 2.65) | 2.37 (1.76, 2.97) | 2.74 (1.80, 3.68) | 3.30 (1.31, 5.29) |
| Total PA | 1.88 (1.46, 2.30) | 2.07 (1.61, 2.52) | 2.08 (1.01, 3.16) | 2.80 (1.52, 4.08) |
| Objective PA | * | 4.21 (2.12, 6.25) | * | 1.56 (-2.85, 5.98) |

CI=confidence interval; PA=physical activity

Fully adjusted model including age, sex, ethnicity, socioeconomic status, employment status, education level, body mass index, smoking status, alcohol consumption, fruit and vegetable, oily fish, non-oily fish, processed meat, red meat intake, and sedentary time.

Leisure-time physical activity

Low physical activity group (<600 MET-mins/week) was the reference group (1)

Moderate physical activity group (600 to <3000 MET-mins/week)

High physical activity group (≥3000 MET-mins/week)

With top-10 comorbidity (n=32,424; deaths=2,023); without top-10 comorbidity (n=456,150; deaths=9,314)

Total physical activity

Low physical activity group (<600 MET-mins/week) was the reference group (1)

Moderate physical activity group (600 to <3000 MET-mins/week)

High physical activity group (≥3000 MET-mins/week)

With top-10 comorbidity (n=28,922; deaths=1,730); without top-10 comorbidity (n=425,717; deaths=8,486)

Objective physical activity

Low physical activity group (4 mins/day of brisk walking) was the reference group (1)

Moderate physical activity group (10 mins/day brisk walking)

High physical activity group (22 mins/day of brisk walking)

With top-10 comorbidity (n=4,396; deaths=53); without top-10 comorbidity (n=91,220; deaths=253)

* Not possible to estimate because of small number of events

**Table S13.** Association between objective physical activity in tertiles of time accumulated above 250 m*g* of total acceleration and mortality

|  | Hazard Ratio (95% CI) | | | |
| --- | --- | --- | --- | --- |
|  | **With multimorbidity** | | **Without multimorbidity** | |
|  | Moderate | High | Moderate | High |
| Hazard ratio (95%CI) |  |  |  |  |
| Model A | 0.36 (0.21, 0.63) | 0.23 (0.10, 0.55) | 0.51 (0.37, 0.72) | 0.27 (0.17, 0.44) |
| Model B | 0.35 (0.20, 0.59) | 0.23 (0.10, 0.54) | 0.46 (0.33, 0.64) | 0.23 (0.15, 0.37) |

CI=confidence interval; PA=physical activity

Lowest PA (4.3 mins/day of brisk walking for exercise was the reference group (1)

Moderate PA (11.5 mins/day of brisk walking for exercise)

High PA (24.5 mins/day of brisk walking for exercise)

With multimorbidity (n=15,607; deaths=106) and without multimorbidity (n=80,009; deaths=200)

Model A was fully adjusted including age, sex, ethnicity, socioeconomic status, employment status, education level, body mass index, smoking status, alcohol consumption, fruit and vegetable, oily fish, non-oily fish, processed meat, red meat intake, and sedentary time.

Model B was calculated using the time of objective physical activity measurements as time zero, adjusted for age at objective physical activity measurements, sex, ethnicity, and socioeconomic status.

# Figure S1. Most to least prevalent chronic conditions used to define multimorbidity (n=491,939)

1. Hypertension 25.9%
2. Asthma 11.6%
3. Cancer 8.2%
4. Depression 5.6%
5. Diabetes 4.2%
6. Angina 3.1%
7. Migraine 2.9%
8. Eczema or dermatitis 2.5%
9. Irritable bowel syndrome 2.3%
10. Myocardial infarction 2.2%
11. Osteoporosis 1.6%
12. Stroke 1.3%
13. Anxiety or panic attacks 1.3%
14. Rheumatoid arthritis 1.1%
15. Glaucoma 1.1%
16. Epilepsy 0.8%
17. Vestibular disorder 0.8%
18. Atrial fibrillation 0.7%
19. Chronic sinusitis 0.6%
20. Tuberculosis 0.5%
21. Meningitis 0.4%
22. Chronic obstructive pulmonary disease 0.3%
23. Multiple sclerosis 0.3%
24. Thyroid problem 0.3%
25. Parkinson’s disease 0.2%
26. Prostate problem 0.2%
27. Bronchiectasis 0.2%
28. Anaemia 0.2%
29. Peripheral vascular disease 0.2%
30. Heart failure 0.1%
31. Cirrhosis 0.1%
32. Hepatitis 0.1%
33. Schizophrenia 0.1%
34. Dementia 0%
35. Chronic kidney disease 0%
36. Inflammatory bowel disease 0%

Prevalence

**(+)**

**(-)**

# Figure S2. Association between self-reported physical activity in tertiles and mortality

CI=confidence interval; M=middle tertile for leisure-time physical activity (332 to 1050 MET-min/week), and for total physical activity (1013-<2628 MET-min/week); U=upper tertile for leisure-time physical activity (≥1050 MET-min/week), and for total physical activity (≥2628 MET-min/week).

Lowest tertile for leisure-time physical activity (<332 MET-min/week), and total physical activity (<1013 MET-min/week) was the reference group (1).

P-value for trend was P<0.001 for all models.

Model 1 was unadjusted.

Model 2 was adjusted for age and sex.

Model 3 was adjusted for age, sex, ethnicity, socioeconomic status, employment status and education level.

Model 4 was adjusted for all variables in model 3 plus body mass index, smoking status, alcohol consumption, fruit and vegetable, oily fish, non-oily fish, processed meat, red meat intake, and sedentary time.

Leisure-time physical activity: with multimorbidity (n=95,617; deaths=4,326), and without multimorbidity (n=392,959; deaths=7,011).

Total physical activity: with multimorbidity (n=86,598; deaths=3,758), and without multimorbidity (n= 368,043; deaths=6,458).

# Figure S3. Association between objective physical activity and mortality, taking the time at objective PA assessment as the start of the follow-up in participants with and without multimorbidity

CI=confidence interval; M=moderate physical activity group (10 mins/day brisk walking); H= high physical activity group (22 mins/day of brisk walking).

Low physical activity group (4 mins/day of brisk walking) was the reference group (1).

P-value for trend was P<0.001 for all models.

Model 1 was unadjusted, Model 2 was adjusted for age at objective at the physical activity measurements and sex, Model 3 was adjusted for age at objective physical activity measurements, sex, ethnicity, and socioeconomic status.

Objective physical activity: with multimorbidity (n=15,607; deaths=106) and without multimorbidity (n=80,009; deaths=200).
